# Protein Profile of Blood Monocytes is Altered in HTLV-1 Infected Patients: Implications for HAM/TSP Disease

**DOI:** 10.1038/s41598-018-32324-2

**Published:** 2018-09-25

**Authors:** Juliana Echevarria-Lima, Denise de Abreu Pereira, Thais Silva de Oliveira, Otávio de Melo Espíndola, Marco Antonio Lima, Ana Cláudia Celestino Leite, Vanessa Sandim, Clarissa Rodrigues Nascimento, Dario E. Kalume, Russolina B. Zingali

**Affiliations:** 1 Lab. de Imunologia Básica e Aplicada, Depto. of Immunology, Instituto de Microbiologia Paulo de Góes, Universidade Federal do Rio de Janeiro (UFRJ), Rio de Janeiro, RJ Brazil; 2 Unidade de Espectrometria de Massas e Proteômica (UEMP), Instituto de Bioquímica Médica Leopoldo de Meis and Instituto Nacional de Biologia Estrutural e Bioimagem (INBEB), UFRJ, Rio de Janeiro, RJ Brazil; 3 Programa de Oncobiologia Celular e Molecular, Coordenação Geral de Ensino e Pesquisa, Instituto Nacional de Câncer, Rio de Janeiro, RJ Brazil; 4 Lab. de Pesquisa Clínica em Neuroinfecções, Instituto Nacional de Infectologia Evandro Chagas (INI), Fundação Oswaldo Cruz (Fiocruz), Rio de Janeiro, RJ Brazil; 5 Lab. de Imunologia Molecular, Instituto de Biofísica Carlos Chagas Filho (IBCCF), UFRJ, Rio de Janeiro, Brazil; 6 Lab. Interdisciplinar de Pesquisas Médicas, Instituto Oswaldo Cruz (IOC), Fundação Oswaldo Cruz (Fiocruz), Rio de Janeiro, RJ Brazil

## Abstract

Human T-cell lymphotropic virus type-1 (HTLV-1) is the etiological agent of HTLV-1-associated myelopathy/tropical spastic paraparesis (HAM/TSP). The endothelial breakdown and migration of leukocytes, including monocytes, to the spinal cord are involved in HAM/TSP development. Monocytes from HTLV-1-infected individuals exhibit important functional differences when compared to cells from uninfected donors. Using proteomic shot gun strategy, performed by nanoACQUITY-UPLC system, we analyzed monocytes isolated from peripheral blood of asymptomatic carriers (AC), HAM/TSP and uninfected individuals. 534 proteins were identified among which 376 were quantified by Expression^E^ software. Our study revealed a panel of changes in protein expression linked to HTLV-1 infection. Upregulation of heat shock proteins and downregulation of canonical histone expression were observed in monocytes from HTLV-1-infected patients. Moreover, expression of cytoskeleton proteins was increased in monocytes from HTLV-1-infected patients, mainly in those from HAM/TSP, which was confirmed by flow cytometry and fluorescence microscopy. Importantly, functional assays demonstrated that monocytes from HAM/TSP patients present higher ability for adhesion and transmigration thought endothelium than those from AC and uninfected individuals. The major changes on monocyte protein profile were detected in HAM/TSP patients, suggesting that these alterations exert a relevant role in the establishment of HAM/TSP.

## Introduction

Human T-cell lymphotropic virus type 1 (HTLV-1) is the etiological agent of adult T-cell leukemia/lymphoma (ATLL) malignancy^1^ and HTLV-1-associated myelopathy/tropical spastic paraparesis (HAM/TSP), a chronic progressive disabling disease characterized by demyelination, axonal loss, neuronal degeneration and gliosis^2^. The neurodegeneration affects mainly the thoracic spinal cord section, which leads to slowly progressive spastic paraparesis with low back pain, in addition to bowel, urinary and sexual dysfunctions^3,4^. About 5–10% of total infected population, referred as symptomatic patients, develop either ATLL or HAM/TSP^5^; however, some studies have shown that HTLV-1-asymptomatic carriers (AC) present important clinical manifestations, such as paresthesia, paresis, nocturia, arthralgia, gingival bleeding, erectile dysfunction and opportunistic infections^6,7^. High prevalence areas include Latin America, Southern Japan, Central and Western Africa^7^. Brazil is the largest endemic country for HTLV-1 infection, and its prevalence among blood donors ranges from 0.04 to 1%^7^.

CD4^+^ T lymphocytes are the predominant target for HTLV-1 infection^8,9^. HTLV-1-infected T lymphocytes display higher levels of spontaneous proliferation and activation markers than their uninfected counterparts^10,11^. HAM/TSP disease depends on endothelial breakdown and migration of activated and/or infected T lymphocytes into the spinal cord^12,13^. Analyses of lesions from HAM/TSP patients revealed the presence of infected CD8^+^ T cells, B cells^14^, dendritic cells (DCs)^15^, macrophages^16^ and monocytes^14,16–18^, suggesting they may play a role during disease onset.

Monocytes constitute a multifunctional heterogeneous cell population associated with immune response, tissue repair and homeostasis^19^. HAM/TSP patients present lower frequencies of classical monocytes than uninfected individuals^20,21^. Our group demonstrated that monocytes from HTLV-1-infected individuals, AC and HAM/TSP patients, presented reduced ability for *in vitro* differentiation into DCs when compared to monocytes from uninfected donors^22^. However, HTLV-1 proviral load in freshly isolated monocytes was not associated with this finding, suggesting that impaired DC differentiation reflects alterations other than direct infection of monocytes^22^. Here we investigated the hypothesis that monocytes from HTLV-1-infected patients could exhibit distinct molecule profiles, thus contributing to alteration of their phenotype and function.

HTLV-1-infected cells have been described as exhibiting alterations in protein expression profiles, such as the reduction in choline, phosphocholine, spermine and glutathione production, and an increase in creatine, dopamine, arginine and adenosine monophosphate levels^23^.

For the first time, monocytes obtained from AC peripheral blood, HAM/TSP and uninfected individuals were studied using proteomic analysis. Proteomic analysis is a powerful tool to understand HTLV-1 pathophysiology, indicating candidate biomarkers for diagnosing, staging, and tracking HTLV-1-associated diseases, and our results evince that HTLV-1 infection impact monocyte protein profile during HAM/TSP disease.

## Materials and Methods

### Subjects

This study enrolled HAM/TSP patients and AC (from a cohort attending the HTLV-1 outpatient clinic at Instituto Nacional de Infectologia Evandro Chagas (INI/FIOCRUZ–RJ, Brazil), and uninfected individuals as a control group (Table 1). HAM/TSP was diagnosed according to the WHO criteria. All experimental procedures were performed in accordance to the Brazilian Resolution 196/96 and 466/12 of the National Health Council published by the Brazilian National Research Ethics Commission (Comissão Nacional de Ética em Pesquisa/CONEP). The Ethics Committee of the Hospital Universitário Clementino Fraga Filho (RJ, Brazil) approved the study protocol (CAAE-number_04203212.8.0000.5262), and peripheral blood was collected after informed consent was obtained, and peripheral blood was collected after informed consent.

**Table 1:** Donors.

Subject	N°	Duration of illness (Mean ± SD; years)	Clinical status	Proviral load
NI	15	n.d.	n.d.	n.d.
AC	17	16.9 ± 3.3	no disability	3.76 ± 3.49
HAM/TSP	16	12.3 ± 5.9	spastic paraparesis (12) wheelchair use (4)	10.70 ± 3.35

NI (uninfected donors); AC (asymptomatic); HAM/TSP (HTLV-1-associated myelopathy/tropical spastic paraparesis); n.d. (undetermined). Proviral load = number of HTLV-1-infected cells/100 leukocytes.

### Purification of monocytes

Peripheral blood mononuclear cells (PBMC) were isolated using Ficoll-Paque density gradient centrifugation (Sigma-Aldrich), and then washed three times with phosphate buffer saline (PBS). Monocytes were purified by negative selection using Dynabeads Untouched Human Monocytes Kit (Invitrogen). After that, cells were washed with PBS and treated with lysis solution for LC-MSE supplemented with protease inhibitor cocktail tablets (Roche). Extracts were sonicated for 5 minutes, centrifuged at 15,000 × g for 15 minutes at 4 °C, and the supernatants were recovered. Protein concentration for each extraction was determined by BCA method (Sigma-Aldrich), and samples were stored at −80 °C.

### Sample preparation and protein extract digestion

Eight samples from each group (AC, HAM/TSP and uninfected) were pooled before protein digestion. Following four times buffer exchange to 50 mM NH_4_HCO_3_ using 3-kDa cutoff Amicon filter (Millipore), the protein extract supernatant samples were quantified. Monocyte lysate supernatant (2 µg/μL) in 50 mM NH_4_HCO_3_ was mixed to *Rapi*GEST 0.2% (w/v) (Waters) and incubated at 80 °C for 15 minutes.Samples were reduced by the addition of dithiothreitol (100 mM) at 60 °C for 30 minutes, followed by incubation with iodoacetamide solution (300 mM) at room temperature (RT) for 30 minutes. Digestion was performed with porcine trypsin (Promega, 1:100) at 37 °C overnight. To precipitate and remove the surfactant *Rapi*GEST and stop digestion, 5% TFA (v/v) solution was added. Samples were vortexed, incubated for 90 minutes at 37 °C, and then centrifuged at 6 °C for 30 minutes. Supernatants were dried down in vacuum centrifuge^24^, and pellets were resuspended in anion exchange column loading buffer (5 mM NH_4_HCO_2_, 5% CH_3_CN (v/v) pH 3.2). Aliquots of each sample were transferred to Waters Total Recovery vials and injected onto a nanoUPLC system coupled to Synapt HDMS mass spectrometer (Waters).

### Label-free 2D LCMS^E^

Nanoscale on-line LC separation of tryptic peptides from the monocyte lysate was performed using nanoACQUITY UPLC system (Waters) equipped with strong cation-exchange (SCX) column (nanoACQUITY UPLC SCX TRAP Column, 180 µm × 23.5 mm) (Waters), pre-column (Symmetry C18 5 µm, 180 µm × 20 mm) (Waters) and analytical reversed-phase column (BEH 130 C18 Column 1.7 µm, 75 µm × 150 mm) (Waters). The pooled samples from each group (8 µg) were initially injected by an auxiliary nanoACQUITY system pump (ASM) onto SCX column using loading buffer solution with a 5 µL/minute flow rate for 10 minutes. After injection, a step gradient was performed by loading 9 µL of 8 salt plugs onto SCX columns. The plug solutions varied from 50 to 350 mM NH_4_HCO_2_ and 5 to 50% (v/v) acetonitrile according to Silva *et al*.^25^.

The peptides were separated onto the analytical column using linear gradient with a 300 nL/minute flow rate. Mobile phase A was 0.1% (v/v) formic acid in water, while B was 0.1% (v/v) in acetonitrile. The gradient consisted of 5 to 50% mobile phase B for 57 minutes, followed by an increase to 85% for 3 minutes, and rinse with 85% during 3 minutes. Then mobile phase B decreased from 85% to 5% in 3 minutes, and the column was finally re-equilibrated to the initial condition for 15 minutes. Analytical column temperature was kept at 35 °C.

Mass spectrometer (MS) was operated in “V mode” with typical resolving power of at least 10,000 full-width half-maximum (FWHM). All analyses were performed in positive nanoelectrospray ion mode. TOF mass analyzer was calibrated with a 100 fmol glu-fibrinopeptide (GFP) solution delivered from ASM using a constant 600 nL/minute flow rate to the reference sprayer of NanoLock Spray source with a 30-seconds frequency. GFP fragment ions from m/z 50 to 2000 and double-charged precursor ion [M^+^ 2 H]2^+^  = 785.8426 were used as lock mass correction for post-acquisition data calibration. For nano LC-MS^E^ analysis acquisition mode, MS data were collected by alternating low-energy (at constant collision energy of 4 eV) and elevated-energy (ramped from 15 to 55 eV) conditions. Continuum spectra acquisition time, in each mode, was 0.8 s with a 0.02 s interscan delay.

### LCMS^E^ data processing, protein identification and differential expression analysis

MS data obtained from LCMS^E^ were processed by ProteinLynx Global Server (PLGS) 2.5.1 (Waters). The embedded IdentityE algorithm identified proteins, and data searches were performed against Human UniProt database (released July_2013). Randomized sequences were appended to the original database for estimating false-positive discovery rate. The following parameters were selected for protein searches: one missed trypsin cleavage site; cysteine carbamidomethylation as fixed modification; N-terminal acetylation; asparagine and glutamine deamidation and methionine oxidation were considered variable modifications. Precursor and fragment ion tolerances were determined automatically as 10 and 20 ppm, respectively. We considered as positive protein identification match the detection of at least three fragment ions per peptide, seven fragment ions per protein, and one peptide per protein. False-positive discovery rate was set at a maximum of 1% according to the established search algorithm^26^. For assessment of differential expression of the protein among uninfected, AC and HAM/TSP pooled samples, three independent 2D-LCMS^E^ runs were performed. Final protein output tables from all replicates generated by ion accounting algorithm integrated in PLGS were merged by MassPivot 2.2.3^27^ used to estimate the constitutive protein that presented no significant difference in relative abundance, and the lowest coefficient of variation (CV) of the three most intense ions (top3) in all replicates. Normalization of the total amount of proteins was based on LogE intensity of exact mass retention time (EMRT) clusters according to Silva *et al*. (2005)^28^ using ExpressionE embedded in PLGS. To analyse quality control of LCMS^E^ data TIBCO Spotfire program was used. Scaffold (v. 3.3.2) was used to remove redundancy identifications and to analyse the quantitative protein profile of each pool of samples by Venn diagram. The ID confidence range used was over 95%. Gene ontology classification was performed with Panther 8.0^29^.

### Fluorescence microscopy

To ensure validation of the data, PBMC obtained from individuals other than those who composed the monocyte pool of the proteomic analysis were used in the fluorescence microscopy experiments. PBMCs were plated in glass coverslips at 5 × 10^6^ cells/well in RPMI-1640 medium supplemented with 10% FBS, penicillin and streptomycin (Gibco) for 2 h at 37 °C under 5% CO_2_ in a humidified atmosphere. Non-adherent cells were removed by extensive washing and fixed in 2% paraformaldehyde (Sigma-Aldrich) overnight at 4 °C. Cells were permeabilized with permeabilization buffer (eBioscience) for 30 minutes at RT; then, washed and labeled with rabbit anti-human-gelsolin (1:100; v/v) (SantaCruz Biotechnology) or anti-human-histone-2B antibody (1:300; v/v) (Cell Signaling Technology) overnight at 4 °C. Cells were washed and stained with anti-rabbit-IgG Alexa647 conjugated (1:500; v/v) or with anti-rabbit-IgG Alexa488 conjugated antibody (1:500; v/v) (Life Technologies). After 1 h at RT, cells were washed and stained with phalloidin Alexa Fluor488 (1:20 v/v; Molecular Probes) at RT for 30 minutes. Cells were washed and labeled with 4′,6-diamidino-2-phenylindole (DAPI - 2 mg/mL; Sigma-Aldrich). Coverslips were rinsed and mounted with Fluoromount-G medium (eBioscence). Cell images were captured through microscopy (Leica TCS-SP5), using 63X magnification objectives. Image analysis was performed by ImageJ_v1.46.

### Flow cytometry

Monocytes from individuals other than those who composed the monocyte pool of the proteomic analysis were used in the flow cytometry experiments. 5 × 10^5^ cells/mL were washed with PBS + FBS 5% and labeled with monoclonal antibody anti-human-HLA-DR APC (1:10; v/v) (Becton & Dickinson; BD) at 4 °C. After 30 minutes, cells were washed, fixed with 2% paraformaldehyde and permeabilized for 30 minutes at RT. Cells were washed and stained with rabbit anti-human-gelsolin antibody (1:100; v/v) overnight at 4°C. Thereafter, cells were washed and labeled with anti-rabbit-IgG Alexa488 conjugated antibody (1:500; v/v) for 30 minutes at RT. Cells were washed, and the fluorescence was examined using FACSCalibur (BD). Monocytes were analyzed after gating based on forward versus side scatter and HLA-DR expression. Analysis was performed using Summit V4.0.

### Monocyte adhesion and transmigration

Human Brain Microvascular Endothelial Cells (hBMECs) were seeded on plate wells at a concentration of 3 × 10^5^ cells/mL. The monocytes were obtained from individuals other than those who composed the monocyte pool of the proteomic analysis were used in the adhesion and transmigration assays. Monocytes were labelled with 1 µM CFSE (Invitrogen) for 15 minutes, washed three times and resuspended in medium supplemented with 10% FCS. 2 × 10^5^ cells were transferred onto hBMECs monolayers and incubated for 3 h at 37 °C under 5% CO_2_ in humidified atmosphere. Adherent cells were quantified by lysing cells with 0.1 M NaOH and measuring the fluorescence intensity using SpectraMax Paradigm reader (Molecular Devices). Data are expressed as mean ± SEM of arbitrary units.

Monocyte transmigration assays were performed using Millipore Millicell inserts (12 mm diameter, 8 µm pore size). hBMEC were seeded on the upper side of the filter at a concentration of 3 × 10^5^ cells/insert, and monolayers reached confluence. 2 × 10^5^ CFSE-labelled monocytes were transferred onto monolayers and incubated for 12 h at 37 °C under 5% CO_2_ in humidified atmosphere. Transmigrated monocytes were recovered and counted by flow cytometer using 40,000 Flow-Check Fluorospheres (Beckman Coulter).

### Detection of HTLV-1 infection and HTLV-1 proviral load quantification

DNA was extracted from monocytes with the QIAamp DNA blood mini kit (Qiagen), and DNA was eluted in 30 µl. HTLV-1 proviral load was determined by quantitative PCR in a Rotor-Gene Q instrument (Qiagen), using the Rotor-Gene Probe PCR kit (Qiagen), according to manufacturer’s instructions. Primers and 5′-FAM and 3′-TAMRA-labeled TaqMan® probes (Sigma-Aldrich) for the HTLV-1 *tax* and the human *β-globin* genes, as previously described by Silva *et al*.^30^, were used in independent reactions with 5 µl of DNA. HTLV-1 proviral load was calculated as *tax* copies/(*β-globin* copies/2), and results are shown as infected monocytes per 100,000 cells. Qualitative PCR was performed with 10 µl of DNA in 50 µl reactions using the HotStar Taq Plus PCR kit (Qiagen), following manufacturer’s instructions, using the same primers for HTLV-1 *tax* and human *β-globin* genes. Amplification cycle consisted of: enzyme activation at 95 °C for 5 min, 45 cycles of denaturation at 95 °C for 30 s, annealing at 60 °C for 30 s, and extension at 72 °C for 30 s, and a final extension step at 72 °C for 10 min. PCR products were electrophoresed in 2% agarose gel stained with GelRed® (Biotium) in 1× Tris-Borate-EDTA buffer (Invitrogen) at 100 V for 90 min. Amplification of HTLV-1 *tax* results in a 159 bp PCR product.

### Statistical analysis

Statistical analysis was performed by Mann–Whitney U test, using GraghPad Prism5, and *p*-values ≤0.05 were considered statistically significant.

## Results

### Monocyte proteomic analyzes

As experimental design, we purified monocytes from peripheral blood, chose samples with purity above 95% and pooled them in order to guarantee sufficient material for our analysis. The total amount of protein extract from each pooled monocyte samples varied between 82.9 ± 12.6 µg. Then validation was performed with individual sample. Protein profile of pooled monocytes obtained from peripheral blood of HAM/TSP, AC and uninfected individuals were submitted to tryptic digestion and were analyzed by LCMS^E^, in triplicates. Three replicates data sets were analyzed for each sample group, and prior to performing quantitative analysis. 159,490 EMRT clusters were identified; among the replicate injections, the average CV of the measured signal intensity was under 7% in all three samples. Reproducibility of retention times for the clusters resulted in less than 14%, and they were mostly under 6% (Supplemental Fig. 1A,B), thus indicating excellent reproducibility. For comparing and correlating the intensity of EMRT pairs, diagonal graphics of normalized log EMRT intensity for replicates of two samples were plotted. Binary distribution showed strong correlation as experimental points present an inclination of approximately 45 degrees along a diagonal line, which is close to the ideal plot (Supplemental Fig. 1C). We analyzed data quality generated by searching against human database using PLGS. PepFrag1 and PepFrag2 corresponding to first and second pass in database search algorithm^26^, were estimated as 58.3% and 13.5%, respectively. Moreover, 6.4% of the total peptides were identified as one-missed trypsin cleavage. The other types of peptides matched in the database search were neutral loss (0.7%), variable modifications (15.9%) and in-source fragmentation rate (5.2%). Results obtained for the dynamic range of the experiment including all quantified molecules showed nearly four orders of magnitude, indicating reasonable distribution of high and low molecular mass proteins (Supplemental Fig. 2). To normalize the samples, we used fibrinogen γ-chain, which presented the lowest CV top3 intensity (0.1579) among all replicates.

**Figure 1:**
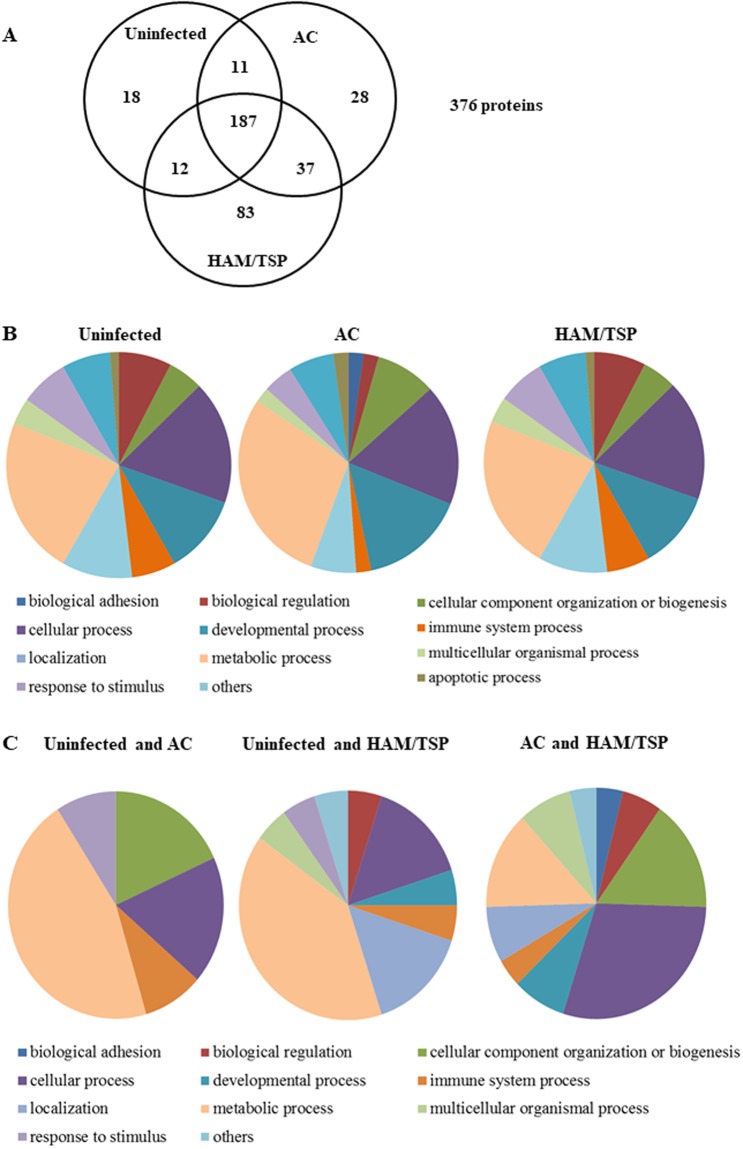
Monocyte proteomic analyses. (**A**) Venn diagram illustrates the 376 quantified proteins distribution among the three pooled monocyte samples derived from AC, HAM/TSP and uninfected individuals. Total proteins were extracted and digested by trypsin followed by 2D label-free LCMS^E^ analysis. (**B**) Functional classification of proteins exclusively expressed in monocytes from uninfected individuals (n = 18), AC (n = 28) or HAM/TSP (n = 83). The quantified proteins were found to be involved in 11 biological processes: adhesion, apoptosis, cellular component organization or biogenesis, cellular process, development, immune system, localization, metabolic, multicellular organismal, regulation, response to stimulus. (**C**) Functional classification of the common proteins that were quantified in two groups of monocytes from: uninfected and AC (n = 11); uninfected and HAM/TSP (n = 12); AC and HAM/TSP (n = 37). The analysis of gene ontology was performed in PANTHER database (version 8.0).

534 proteins were identified by Identity^E^, among which 376 were quantified by Expression^E^. This number of proteins identified is directly related to the filters selected by us in our analysis described above. The distribution of the expressed proteins in Venn diagram showed 187 common proteins expressed in all groups (Fig. 1A and Supplemental Table 1). According to gene ontology classification, the majority of proteins were associated with cytoskeleton (18.62%), nuclear proteins (15.16%), immune response (11.70%), small GTPases (10.11%), heat shock proteins (4.26%) and redox related proteins (3.99%).

Regarding the proteins exclusively expressed in each group, we detected: 18 in samples from uninfected individuals; 28 in samples from AC; and 83 in monocyte samples from HAM/TSP patients (Fig. 1A). Proteins were then subdivided using PANTHER classification system based in biological processes (Fig. 1B). We further analyzed these data as relative frequency distribution (RF) of biological processes to compare the three different groups (Table 2). When comparing monocytes obtained from HAM/TSP patients to those from uninfected individuals, it was observed a decrease in the percentage of proteins related to: cellular component organization or biogenesis (15.62% in uninfected versus 5.06% in HAM/TSP), metabolic processes (31.25% in uninfected versus 22.78% in HAM/TSP), cellular processes (21.87% in uninfected versus 17.72% in HAM/TSP), and apoptosis (3.12% in uninfected versus 1.26% in HAM/TSP). Conversely, we observed an increase in the percentage of proteins related to: localization (3.12% in uninfected versus 10.13% in HAM/TSP), biological regulation (3.12% in uninfected versus 7.59% in HAM/TSP), and immune system processes (3.12% in uninfected versus 6.33% in HAM/TSP).

**Table 2:** Relative frequency (RF) of exclusive proteins identified in monocytes obtained from HAM/TSP, AC and uninfected donors.

Biological Process	RFs		
	Uninfected	AC	HAM/TSP
Apoptotic process	0	2.22	1.26
Biological adhesion	3.12	2.22	0
Biological regulation	15.62	8.89	7.59
Component of cell organization or biogenesis	21.87	17.78	5.06
Cellular process	9.37	15.56	17.72
Developmental process	3.12	2.22	11.39
Immune system process	3.12	6.67	6.33
Localization	31.25	28.89	10.13
Metabolic process	3.12	2.22	22.78
Multicellular organismal rocess	6.25	4.44	3.80
Response to stimulus	3.12	6.67	6.96
Others	3.12	2.22	6.96

Analysis was further extended to proteins identified in both groups of monocytes from uninfected and AC, or from uninfected and HAM/TSP or from AC and HAM/TSP by Identity^E^. Figure 1C shows the number of proteins associated with the biological processes, using PANTHER. Interestingly, the number of proteins commonly expressed in HAM/TSP and AC group was three times higher than those expressed between uninfected and AC individuals, or uninfected and HAM/TSP individuals, which suggests that monocytes from HTLV-1-infected individuals share a particular profile of proteins absent in uninfected individuals. To confirm the differences between monocytes protein profile from uninfected individuals and HTLV-1 infected patients we selected different donors and the analysis was performed individually.

### HSPs are upregulated in monocytes of HTLV-1-infected individuals

**Figure 2:**
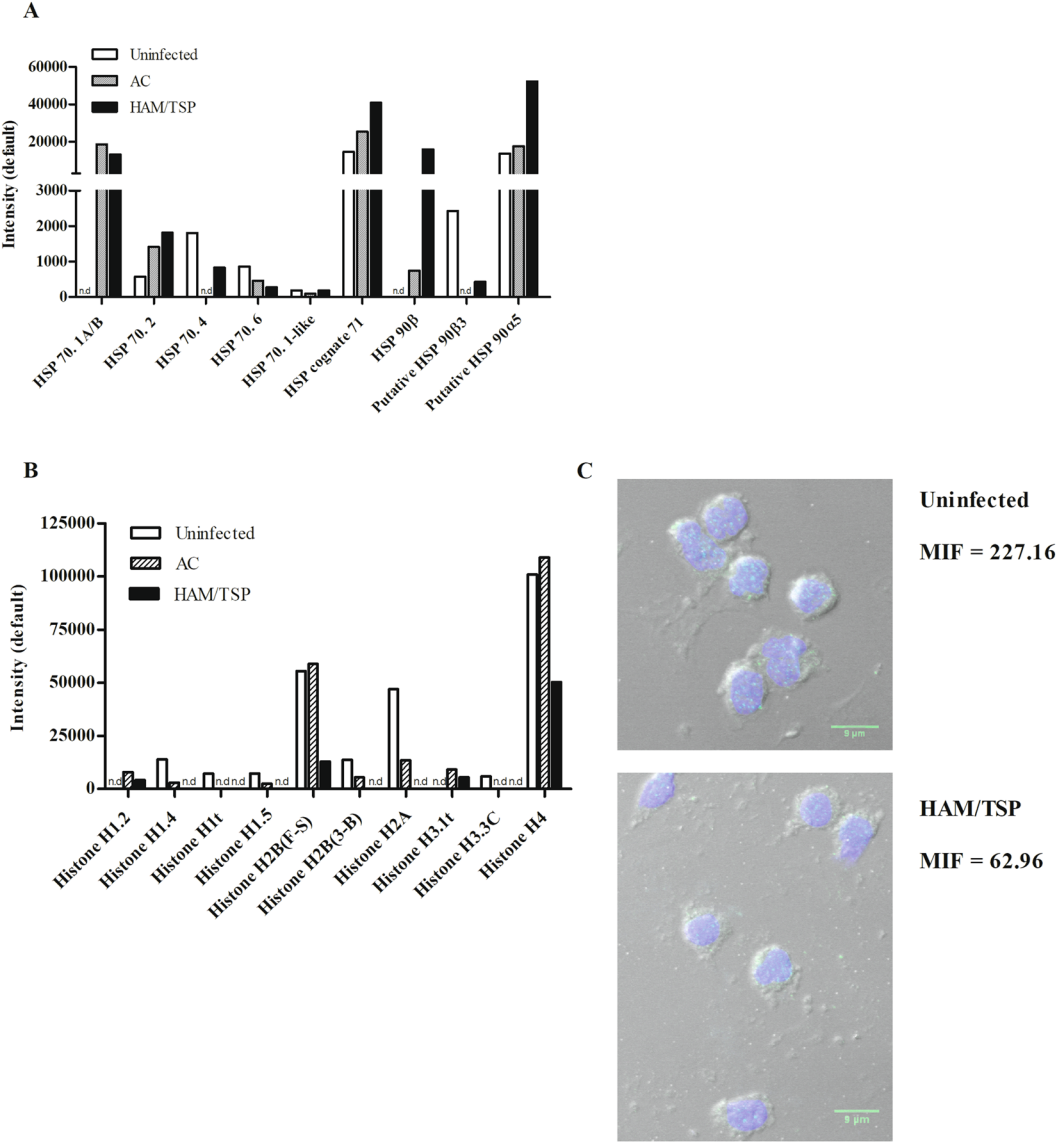
Heat Shock Proteins are upregulated and histone expression is downregulated in monocytes of HTLV-1-infected individuals. The quantification of the results was performed using Scaffold software, based on the precursor intensity quantification approach, which identified and compared the intensities of MS peaks from peptides. (**A**) The graphic represents the intensity values of MS peaks from HSPs of pooled monocyte samples derived from HAM/TSP individuals, AC and uninfected donors. (**B**) The graphic represents the intensity values of MS peaks from histones of monocyte samples derived from HAM/TSP individuals, AC and uninfected donors. n.d. – undetermined. (**C**) Freshly-isolated monocytes were obtained from uninfected donors (n = 3) and HTLV-1-infected patients (n = 3) and fixed in paraformaldehyde. After that, cells were permeabilized, washed and labeled with anti-human-histone-2 antibody overnight at 4 °C. Cells were washed and stained with anti-rabbit-IgG Alexa 488 conjugated antibody. After 1 h at RT, cells were washed and nuclei acid were labeled with DAPI for 2 min at RT. Coverslips were rinsed several times in PBS, drained and mounted with Fluoromount-G medium. Original magnification, 63× objective was visualized under confocal microscopy. MIF indicates the mean of fluorescence intensity. This is a representative result from 3 experiments performed independently.

Once the number of proteins distinctly expressed among the groups were defined, and classified according to biological processes, the expression levels of particular proteins was examined. During virus infection, the expression/activity of chaperone can be modulated. Infection of fibroblasts with human cytomegalovirus, or infection of CD4^+^ T lymphocytes with HIV induces an increase in HSP70 levels^31,32^. Monocytes from HTLV-1-infected individuals expressed increased levels of HSP70-1A/B and 2, HSP90 (β; α5) and HSP70 (4; 6), while HSP90β3 presented decreased levels in comparison to monocytes from uninfected donors (Fig. 2A).

Additionally, we observed altered expression levels of DNA chaperones and High Mobility Group Box (HMGB) proteins. These proteins are associated to replication control, transcription and repair processes. Our data showed that HMGB1 and HMGB2 were more abundant in samples from HTLV-1-infected individuals, markedly in HAM/TSP patients (Supplemental Table 1). According to literature, HMGB1 levels are also increased in ATLL patients and HTLV-1-infected cell lines^33^, which corroborates our findings.

### Histone expression is downregulated in monocytes from HTLV-1-infected individuals

The nucleosome is constituted of two copies of each canonical histone, variants of core histones, linker H1 and non-histone proteins. We identified 43 histones (Supplemental Table 1) characterized as H1-4 subtypes. Peptide intensity analysis showed that histones were detected in lower levels in HTLV-1-infected groups when compared to the uninfected one (Fig. 2B). This downregulation was more pronounced in samples from HAM/TSP patients, in which some histone subtypes were undetectable (H1.4, H1t, H1.5, H2B, H2A, H3.3 C). As H2B was found in the three different groups (at different expression levels), it was chosen to validate the analysis by fluorescence microscopy. To validate our data, monocytes obtained from individuals other than those who composed the monocyte pool of the proteomic analysis were used in the fluorescence microscopy experiments. We observed a decrease in H2B expression in monocytes obtained from HTLV-1-infected individuals when compared to monocytes from uninfected ones (Fig. 2C). This result corroborated data from literature, in which a decrease in histone levels in HTLV-1-infected T cell lines has been reported^34^.

### Different expression of cytoskeleton proteins in monocytes from HTLV-1-infected individuals

**Figure 3:**
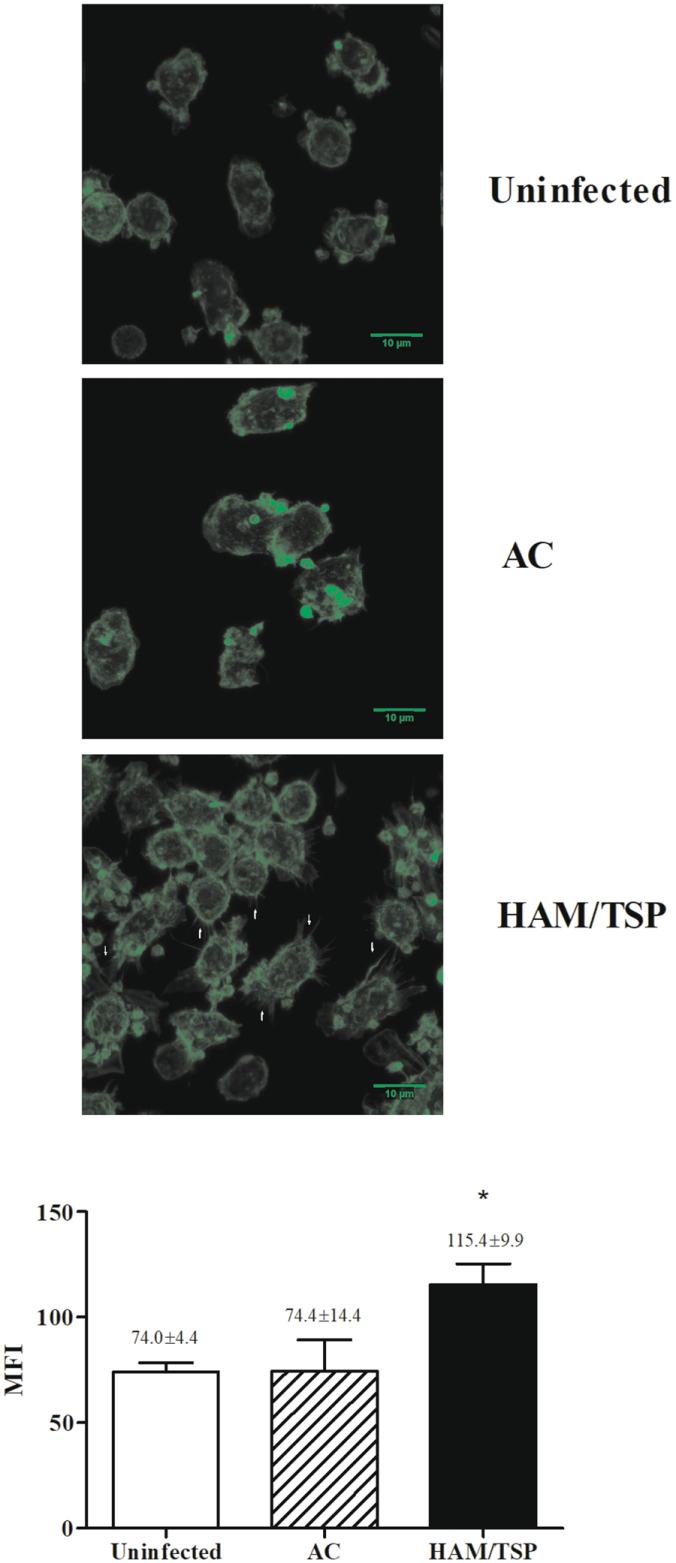
Differential cytoskeleton organization in monocytes from HAM/TSP patients. Freshly-isolated monocytes were obtained from uninfected donors and HTLV-1-infected patients and fixed in paraformaldehyde. After that, cells were permeabilized, washed and labeled with phalloidin Alexa Fluor 488 at RT for 30 min. Coverslips were rinsed several times in PBS, dried and mounted with Fluoromount-G medium. The images are representative of three independent experiments obtained by confocal fluorescence microscopy. Original magnification, 63× objective was visualized under confocal microscopy. The graphic is representative of mean ± SE (values of mean fluorescence intensity – MFI were described into the graphic) of three independent experiments, using cells from NI = 3; AC = 3 and HAM/TSP = 3. The arrows indicate cytoplasmic projections. *Significantly different from the uninfected and AC groups (*p* < 0.05).

**Figure 4:**
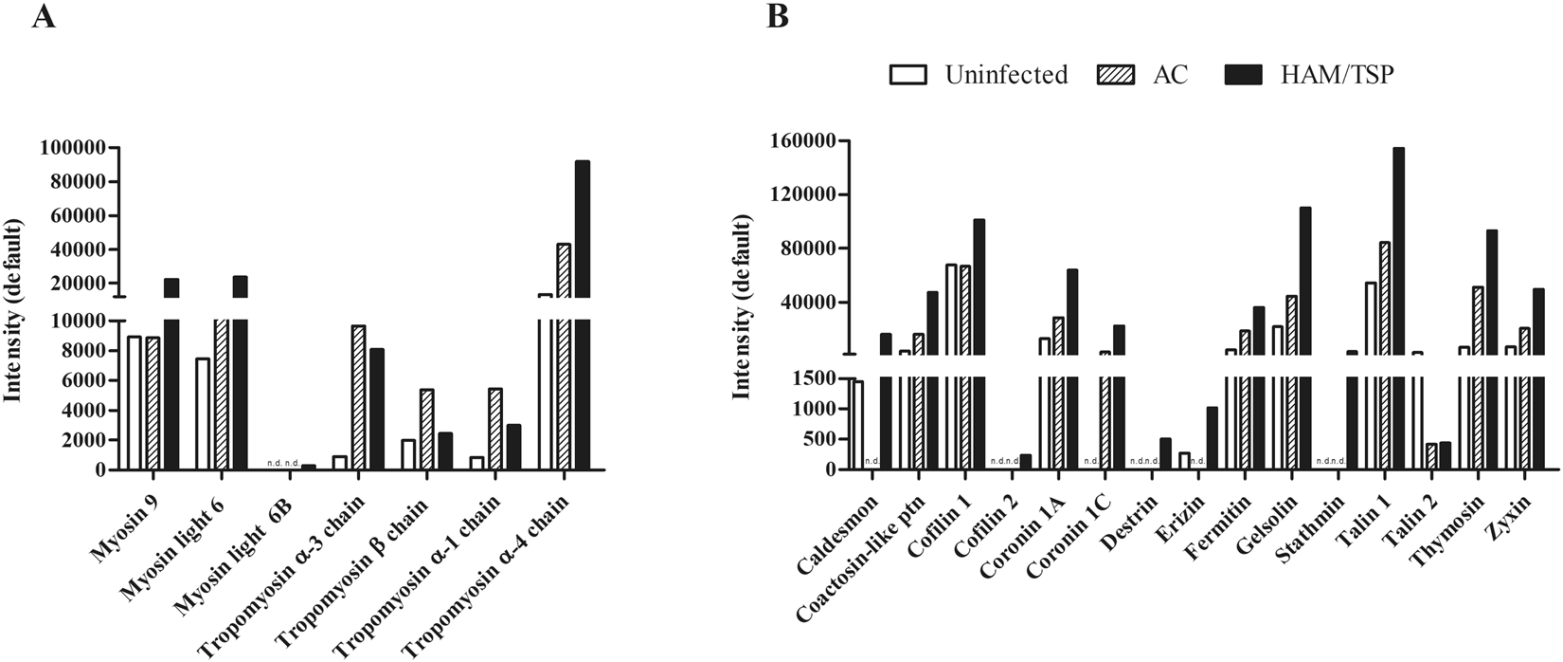
Differential expression of cytoskeleton proteins in monocytes from HTLV-1-infected individuals. Protein quantification was performed using PLGS software, which identified and compared the sum of the three most intensity peptides. (**A**) The graphic represents the intensity values of MS peaks from myosin and tropomyosin of pooled monocyte samples derived from HAM/TSP individuals, AC and uninfected donors. (**B**) The graphic represents the intensity values of MS peaks from other cytoskeleton proteins of pooled monocyte samples derived from HAM/TSP individuals, AC and uninfected donors. n.d. – undetermined.

Since the inspection of monocyte morphology from symptomatic patients in our previous work had revealed alterations consistent with increased adhesion and spreading^22^, here we addressed this issue by analyzing expression levels of cytoskeleton proteins. Our data showed a protein group composed of cytoskeleton components or associated with cytoskeleton dynamics, which were differentially expressed in monocytes from HTLV-1-infected individuals (Supplemental Table 1). Changes in expression levels of actin family proteins and in actin-binding ones were detected. To confirm these actin expression findings, monocytes were stained with phalloidin and analyzed by fluorescence microscopy, using cells obtained from individuals other than those who composed the monocyte pool of the proteomic analysis. Results indicated that actin filaments in monocytes from HAM/TSP individuals presented stronger staining than monocytes from AC or uninfected donors (Fig. 3A). Unlike the rounded shape displayed monocytes from uninfected individuals and AC, monocytes from HAM/TSP individuals (n = 3) presented a distinct pattern for phalloidin staining, which was accompanied by approximately 50% increase in fluorescence (Fig. 3B). As depicted in Fig. 3, the later cells presented thin cytoplasmic projections. In addition, increased levels of myosin and tropomyosin were observed in monocytes derived from HTLV-1-infected patients, and even more striking in those from HAM/TSP patients (Fig. 4A). Moreover, we identified higher levels of those proteins in monocytes from HTLV-1-infected patients, mainly in those from HAM/TSP, when compared to the ones from uninfected donors. Increase in the levels of caldesmon, coactosin-like protein, cofilin 1/2, coronin 1 A/C, destrin, erizin, fermitin, gelsolin, stathmin, talin 1/2, thymosin, and zyxin were also observed in the proteomic analysis of the monocyte samples from HAM/TSP patients (Fig. 4B).

**Figure 5:**
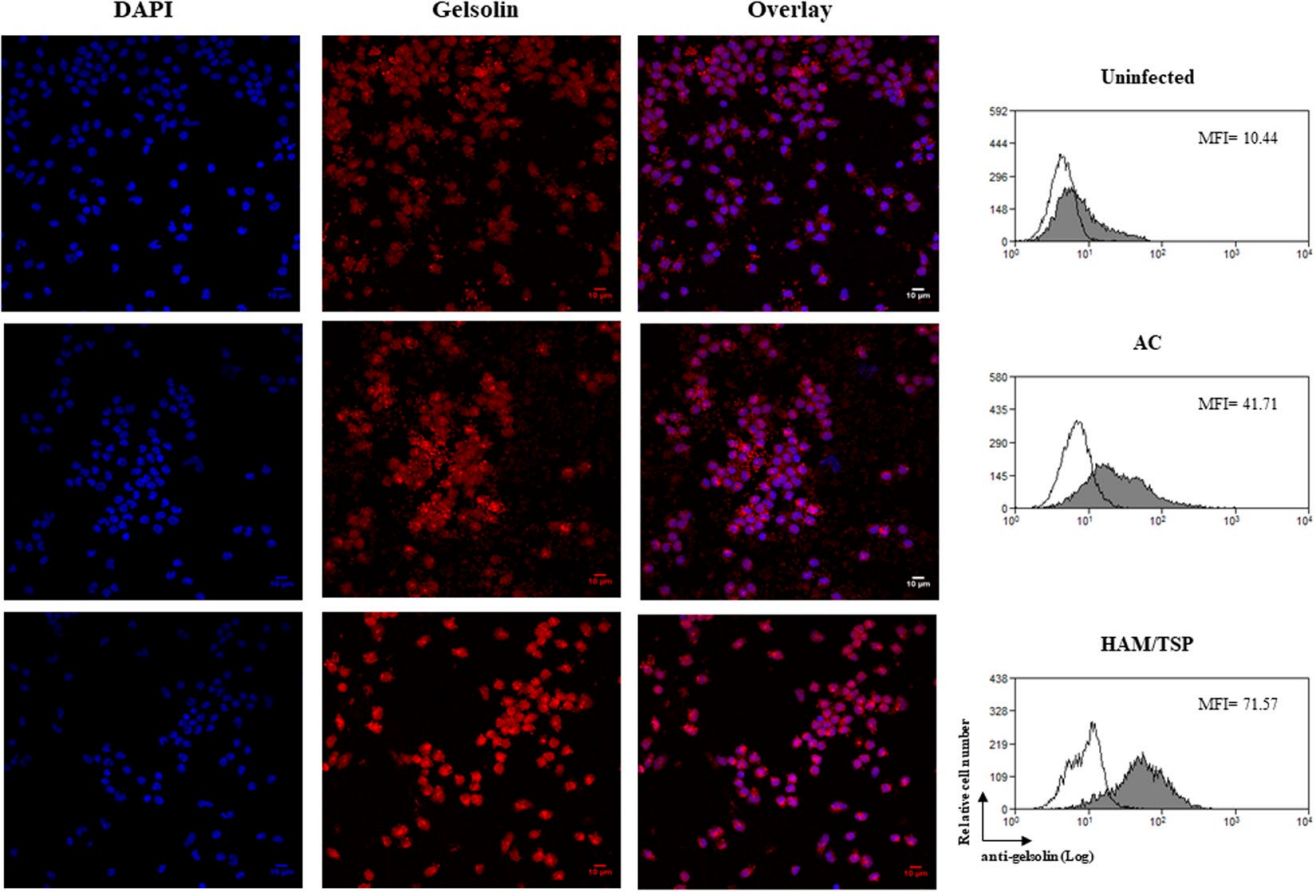
Increased expression of gelsolin in monocytes from HTLV-1-infected individuals. Freshly-isolated monocytes were obtained from uninfected donors and HTLV-1-infected patients and fixed in paraformaldehyde. After that, cells were permeabilized, washed and labeled with anti-human-gelsolin antibody overnight at 4 °C. Cells were washed and stained with anti-rabbit-IgG Alexa 647 (red) conjugated antibody. After 1 h at RT, cells were washed and nuclei acid were labeled with DAPI (blue) for 2 min at RT. Coverslips were rinse several times in PBS, drained and mounted with Fluoromount-G medium. Original magnification, 63× objective was visualized under confocal microscopy. Results are representative of four independent experiments (NI: n = 3; AC: n = 5; HAM/TSP: n = 7). Protein quantification was determined by flow cytometry. Cells were labeled with monoclonal antibody anti-human-HLA-DR APC. Thereafter, cells were washed, fixed and permeabilized. Cells were washed and stained with rabbit anti-human-gelsolin antibody and further washed and labeled with anti-rabbit-IgG Alexa 488 conjugated antibody. Cells were washed and the fluorescence was examined by flow cytometry. The values in the representative histogram are the mean of fluorescence intensity (MFI) of four independent experiments (NI: n = 3; AC: n = 3; HAM/TSP: n = 4). Each experiment was performed with one NI donor, one AC and two HAM/TSP donors.

To validate these data, the expression of gelsolin, a protein associated with migration and adherence, was evaluated by confocal microscopy and flow cytometry. We confirmed that gelsolin expression was higher in monocytes of HTLV-1-infected individuals than in uninfected donors. The median MFI values were more than 400% (mean) higher in monocytes from seven HTLV-1-infected individuals from donors other than those who composed the monocyte pool of the proteomic analysis (Fig. 5).

**Figure 6:**
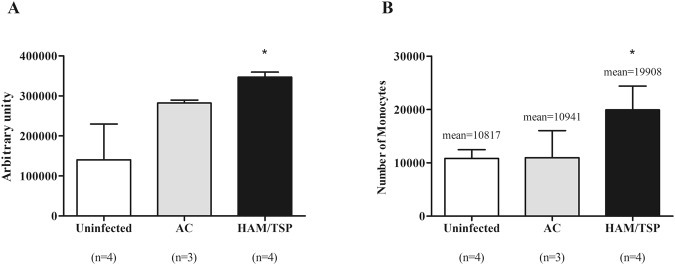
Monocytes obtained from HAM/TSP patients exhibited higher ability of adhesion and transmigration. (**A**) Monocytes were labelled with CFSE, washed and resuspended in RPMI medium supplemented with FCS 10% before transfer to BMEC confluent monolayers. Cells were incubated for 3 h at 37 °C with 5% CO_2_ in humidified atmosphere. Thereafter, quantification of adherent cells was performed by lysing cells with NaOH and measuring the fluorescence intensity. Data are expressed as mean ± SEM of arbitrary units. *Significantly different from the uninfected and AC groups (p < 0.05). The results are representative of four independent experiments. (**B**) Monocyte transmigration assays were performed in 24-well plates using Millipore Millicell inserts. hBMECs were seeded on the upper side and monolayers reached confluence. Monocytes were incubated for 12 h at 37 °C with 5% CO_2_ in humidified atmosphere. In sequence, transmigrated monocytes were recovered and counted by flow cytometer using 40,000 fluorescent beads. *Significantly different from the uninfected and AC groups (*p* < 0.05). Results are representative of five independent experiments, using monocytes from HTLV-1-infected individuals other than those who composed the monocyte pool of the proteomic analysis.

Finally, we investigated whether the modifications in cytoskeleton-related proteins observed in monocytes obtained from HTLV-1-infected patients could be translated into higher ability of adhesion and/or migration. The first step was to compare the adhesion of monocytes to monolayers of hBMECs among the three groups. Monocytes obtained from HAM/TSP patients exhibited higher ability of adhesion to hBMECs than those from AC and uninfected individuals (Fig. 6A), suggesting that overexpression of cytoskeleton proteins might reflect in increased cell adhesion properties. Thereafter, we evaluated if HTLV-1-monocyte cytoskeleton protein profile would impact the migration and adhesion of these cells. Transmigration of monocytes was tested against a confluent monolayer of hBMEC. In Fig. 6B, it is shown that the number of transmigrating cells was at least 2.5-fold higher with monocytes from HAM/TSP patients than those from AC and uninfected individuals. Altogether, these results suggest that monocytes from HAM/TSP individuals are more prone to interact with endothelial cells as well as to transmigrate through endothelium.

### Correlation between HTLV-1 monocyte infection and alterations in protein profile

**Figure 7:**
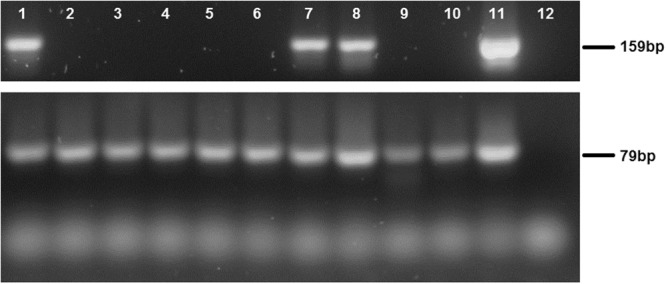
HTLV-1 infection of monocytes. CD14^+^ cells were purified from PBMC derived from HTLV-1-infected patients, and DNA was extracted (Samples 1−10). HTLV-1 provirus was detected by PCR for HTLV-1 *tax* gene (159 bp), and amplification of human β-globin gene (79 bp) was used as internal control. DNA from the HTLV-1-infected MT2 cell line (Sample 11) and water (Sample 12) were respectively used as positive and negative controls.

HTLV-1 may infect monocytes *in vivo*^35^. Thus, we explored whether the monocytes alterations in protein profile and the functions related with these molecules observed was associated to the infection of these cells. The PBMC proviral load in AC was 3.76 ± 3.49 and HAM/TSP was 10.70 ± 3.35. Fresh monocytes were positive for HTLV-1 in 30% of samples from HTLV-1-seropositive individuals and the proviral load was estimated in 3.3 ± 9.97 in one thousand cells (Fig. 7). These findings suggest that the differences observed were influenced by other conditions rather than the infected state of monocytes.

## Discussion

The label-free proteomic approach was used for the first time to characterize and compare the phenotype of monocytes derived from HTLV-1-infected patients and uninfected donors. Results suggested a functional relevance for some differentially expressed proteins, such as chaperones, histones and cytoskeleton proteins. Additionally, results indicated that monocytes derived from AC and HAM/TSP patients present significant similarity in protein profile according to biological processes, suggesting these patients share a particular profile of proteins. However, the monocyte infection was not detected in all samples, suggesting that the protein profile alterations was did not depend on infection.

Tattermusch *et al*. demonstrated using microarray assay, that total population of monocytes from HAM/TSP patients overexpressed IFN-stimulated genes. These genes are involved in several functions including protein–protein interactions and cytoskeleton modulation^36^. It is possible that our findings could be related with lower frequencies of classical monocytes compared to uninfected individuals, but it is important to note that this difference was observed only in HAM/TSP patients^20^. Amorin *et al*. demonstrated that the levels of classical monocytes (CD14^+^CD16^neg^) in total HTLV-1 carries (AC + HAM/TSP) is similar to uninfected individuals, approximately, 84% and 89%, respectively^20^. However, de Castro Amarante *et al*. demonstrated that the frequency of classical monocytes (CD14^+^CD16^neg^) population was reduced in HTLV-1-infected patients^21^.

Monocytes have a plastic nature and pleiotropic functions. *In vivo*, was observed that classical moNnocytes (CD14^+^CD16^neg^) can convert into intermediated monocytes (CD14^+^CD16^+^)^37^. Moreover, both subsets can be differentiated into DCs under inflammatory or non-inflammatory microenvironment^38^. Recently, Hadadi *et al*., demonstrated that classical and intermediated monocytes exhibited a similar secretion of IL-6, IL-8 and TNF-α after LPS stimulation. The authors also observed that the classical and intermediated monocytes produced similar levels of IL-1β and exhibited the same capacity to activate caspase-1 in response to LPS^39^. Although, the HAM/TSP patients present higher frequencies of intermediated monocytes than uninfected individuals^20^, functionality these cells could be the same or very similar HTLV-1 infection. Furthermore, an argument in favor of our data is the significant similarity between monocytes obtained from AC and HAM/TSP patients. Thus, our findings may be related to the monocytes’ role during HTLV-1 infection.

We observed that purified monocytes from HTLV-1-infected individuals express higher levels of HSP70 and HSP90 than monocytes from uninfected donors. Likewise, the HTLV-1-infected monoblastoid cell line also exhibits high levels of HSP70^40^. HSPs are associated to the mechanism of HTLV-1 infection through cell-to-cell contact since anti-HSP70 neutralizing antibody significantly inhibits syncytium formation^41,42^. HSP90 is described as a binding partner of HTLV-1 Tax protein, and the treatment of HTLV-1-infected cells with HSP90 inhibitors repress cell proliferation and induced death^40,43^.

Our results also suggest that HTLV-1 infection might influence the structure of nucleosome. Canonical and variant histone subtypes were downregulated in monocytes derived from HTLV-1-infected individuals, markedly in HAM/TSP patients’ samples. Tax protein promotes downregulation of H1 incorporation into chromatin, leading to activation of histone acetyltransferases, and consequent transcription^44^. Tax was detected in intracellular medium of infected-cell or plasma samples from HAM/TSP and AC patients^45^. Moreover, it was demonstrated that HTLV-1-infected cells can release exosomes containing Tax and viral mRNA transcripts^46^. These data suggest that proteins derived from HTLV-1-infected cells can be delivered to uninfected cells and induce modulation of cell function.

The cellular mechanism that triggers the migration of mononuclear phagocytes to spinal cord is still unknown. Our results demonstrated a differential expression of cytoskeleton proteins in monocytes from HTLV-1-infected individuals. Quantitative protein analyses demonstrated higher levels of actin-binding proteins in monocytes from HTLV-1-infected patients, as well as the cytoskeleton protein gelsolin, mainly in those from HAM/TSP patients. Myosin and tropomyosin were also present in increased levels in monocytes from HAM/TSP patients. We further investigated gelsolin because its active form regulates some functions of actin filaments, including severing, capping, nucleating and phagocytosis^47,48^. Gelsolin expression is altered in cancer, inflammation, idiopathic interstitial pneumonia and Alzheimer’s disease^48,49^. In addition, gelsolin also influences macrophage infiltration to nerve distal to the crush injury site; its absence reduces macrophage infiltration^50^.

Monocytes from HAM/TSP patients exhibited higher ability to adhere to hBMECs monolayers than monocytes from AC and uninfected donors. This phenomenon may be associated to altered cytoskeleton protein expression and HMGB1 production. We also have shown increased levels of HMGB1 in monocytes derived from HTLV-1-infected individuals, mainly in HAM/TSP patients. HMGB1 can be released by activated macrophages and monocytes^51^, and acts as a pro-inflammatory molecule^52^. Furthermore, HMGB1 activates human endothelium, inducing the expression of adhesion molecules^53^. Corroborating this, PBMCs from HAM/TSP patients can adhere with higher affinity to activated endothelium than cells from AC and uninfected individuals^54^.

The Tattermusch *et al*. identified an increase in Tryptophan–tRNA ligase transcripts (*WARS* gene) and IL-15 in monocytes form HAM/TSP^36^. The Tryptophan–tRNA ligase also was found in our samples (Supplemental Table 1), and was related with cytoskeletal reorganization of endothelial cells^55^, but it activity in monocytes remain unknown. For IL-15 it was demonstrated that this cytokine induces a cytoskeleton rearrangement, alternating cell shape and phagocytosis capacity in neutrophils. In monocytes the IL-15 increase the cell adherence through the Small GTPase activation^56^. In our analysis we observed an increase of Small GTPases in AC group (Supplementary Table 1). Together our finds and the literature suggested that the HTLV-1 infection modulated the cytoskeleton organization.

The proteomics and functional results suggest that increased cytoskeleton protein expression can be related to augmented adhesion ability. During neuroinflammation, leukocytes emigrate from peripheral blood to the CNS across the blood-brain-barrier, which depends on cell adhesion to the microvasculature, followed by crawling and transmigration^57,58^. During HIV infection, monocyte trafficking across the blood-brain-barrier depends on cytoskeletal modifications, including increased levels of cortactin^58^. Cortactin was detected only in monocytes from HAM/TSP. It contributes to the organization of the actin cytoskeleton and cell structure and plays a role in the regulation of cell migration, corroborating our functional data. Our results indicate that alterations in cytoskeleton protein profile in monocytes from HAM/TSP patients might be involved in transmigration to spinal cord.

In conclusion, this study was the first to demonstrate that monocytes obtained from HTLV-1-infected patients exhibit distinct protein profiles compared to cells from uninfected individuals. Our results suggest that during HTLV-1 infection the altered environment induces profound modifications that can contribute to: alteration in immune response that makes HTLV-1 carriers more susceptible to opportunistic infections; impairment of DC differentiation in HTLV-1-infected patients; presence of mononuclear phagocytes in spinal cord lesions and development of neurological disease. This study points a new perspective to researches involving a *cohort* of HTLV-1-infected patients, exploring alterations in cytoskeleton protein expression as biomarker for neuroinflammation.

## Electronic supplementary material

Supplementary information
