# Supplementary material for: Protein Profile of Blood Monocytes is Altered in HTLV-1 Infected Patients: Implications for HAM/TSP Disease
Source: Sci Rep. 2018 Sep 25;8:14354. doi: 10.1038/s41598-018-32324-2 (PMC6156329; doi:10.1038/s41598-018-32324-2)
Supplement: Supplementary file 1 — Supplementary information [file 41598_2018_32324_MOESM1_ESM.pdf]

Protein Profile of Blood Monocytes is Altered in HTLV-1 Infected Patients: Implications for HAM/TSP Disease

Monocytes and HAM/TSP Disease
Major Article

Juliana Echevarria-Lima,[1]* Denise de Abreu Pereira,[2,3] Thais Silva de Oliveira,[1] Otávio de Melo Espíndola,[4] Marco Antonio Lima,[4] Ana Cláudia Celestino Leite,[4] Vanessa Sandim Siqueira,[2] Clarissa Rodrigues Nascimento,[5] Dario E. Kalume[6] and Russolina B. Zingali[2]

[1]Lab. de Imunologia Básica e Aplicada, Depto. of Immunology, Instituto de Microbiologia Paulo de Góes, Universidade Federal do Rio de Janeiro (UFRJ), RJ, Brazil;
[2]Unidade de Espectrometria de Massas e Proteômica (UEMP), Instituto de Bioquímica Médica Leopoldo de Meis and Instituto Nacional de Biologia Estrutural e Bioimagem (INBEB), UFRJ, RJ, Brazil;
[3]Programa de Oncobiologia Celular e Molecular, Coordenação Geral de Ensino e Pesquisa, Instituto Nacional de Câncer, RJ, Brazil;
[4]Lab. de Pesquisa Clínica em Neuroinfecções, Instituto Nacional de Infectologia Evandro Chagas (INI), Fundação Oswaldo Cruz (Fiocruz), RJ, Brazil;
[5]Lab. de Imunologia Molecular, Instituto de Biofísica Carlos Chagas Filho (IBCCF), UFRJ; [6]Lab. Interdisciplinar de Pesquisas Médicas, Instituto Oswaldo Cruz (IOC), Fiocruz, Rio de Janeiro, RJ, Brazil.
*Corresponding author: juechevarria@micro.ufrj.br, Juliana Echevarria Lima, PhD, Lab. de Imunologia Básica e Aplicada, Depto. of Immunology, Instituto de Microbiologia Paulo de Góes, CCS, Sala I2-43, Universidade Federal do Rio de Janeiro. Av. Carlos Chagas Filho, 373. CEP: 21941-590 - Rio de Janeiro, RJ, Brazil. Tel. +55 213938-6748.

Accession	Description	Score	Unique	Ratio CTR:AC	Log(e) Ratio CTR:AC	*P Value (CTR:AC)	Ratio HAM/TSP :AC	Log(e) Ratio HAM/TSP: AC	*P Value (HAM/TSP: AC)	Ratio HAM/TSP :CTR	Log(e) Ratio HAM/TSP: CTR	*P value HAM/TSP :CTR
1433B_HUMAN	14 3 3 protein beta alpha YWHAB 1 3	4895,61	-	0,94	-0,06	0,30	0,93	-0,07	0,29	0,99	-0,01	0,44
1433E_HUMAN	14 3 3 protein epsilon YWHAE 1 1	9434,37	-	0,76	-0,28	0,02	0,86	-0,15	0,12	1,14	0,13	0,88
1433F_HUMAN	14 3 3 protein eta YWHAH 1 4	2877,92	-	0,77	-0,26	0,02	0,78	-0,25	0,00	1,02	0,02	0,52
1433G_HUMAN	14 3 3 protein gamma YWHAG 1 2	2882,73	-	0,79	-0,23	0,05	0,77	-0,26	0,04	0,98	-0,02	0,42
1433S_HUMAN	14 3 3 protein sigma SFN 1 1	2467,11	-	0,81	-0,21	0,04	0,76	-0,28	0,01	0,93	-0,07	0,30
1433T_HUMAN	14 3 3 protein theta YWHAQ 1 1	2491,02	-	0,77	-0,26	0,08	0,84	-0,18	0,08	1,08	0,08	0,69
1433Z_HUMAN	14 3 3 protein zeta delta YWHAZ 1 1	15812,50	-	0,73	-0,31	0,00	1,23	0,21	1,00	1,68	0,52	1,00
6PGD_HUMAN	6 phosphogluconate dehydrogenase	1352,70	-	1,36	0,31	0,86	1,15	0,14	0,69	0,84	-0,17	0,24
RL5_HUMAN	60S ribosomal protein L5 RPL5 1 3	206,69	AC	AC	AC	AC	AC	AC	AC	-	-	-
GRP78_HUMAN	78 kDa glucose regulated protein HSPA5 1 2	2380,61	-	0,88	-0,13	0,18	1,23	0,21	0,99	1,40	0,34	1,00
ACTC_HUMAN	Actin alpha cardiac muscle 1 ACTC1 1 1	25161,54	-	0,89	-0,12	0,00	1,08	0,08	0,97	1,22	0,20	1,00
ACTS_HUMAN	Actin alpha skeletal muscle ACTA1 1 1	25049,32	-	0,85	-0,16	0,00	1,07	0,07	0,95	1,26	0,23	1,00
ACTA_HUMAN	Actin aortic smooth muscle ACTA2 1 1	24295,33	-	0,89	-0,12	0,01	1,08	0,08	0,97	1,21	0,19	1,00
ACTB_HUMAN	Actin cytoplasmic 1 ACTB 1 1	45931,79	-	0,84	-0,18	0,00	0,32	-1,13	0,00	0,39	-0,95	0,00
ACTG_HUMAN	Actin cytoplasmic 2 ACTG1 1 1	61740,30	-	0,86	-0,15	0,00	0,93	-0,07	0,06	1,08	0,08	0,94
ACTH_HUMAN	Actin gamma enteric smooth muscle ACTG2 1 1	24295,33	-	0,90	-0,11	0,01	1,07	0,07	0,98	1,21	0,19	1,00
ARC1B_HUMAN	Actin related protein 2 3 complex subunit 1B ARPC1B 1 3	1220,67	-	0,79	-0,23	0,17	0,53	-0,63	0,01	0,67	-0,40	0,08

ARPC2_HUMAN	Actin related protein 2 3 complex subunit 2 ARPC2 1 1	513,28	-	AC	AC	AC	0,79	-0,24	0,14	HAM/TSP	HAM/TSP	HAM/TSP
ARPC3_HUMAN	Actin related protein 2 3 complex subunit 3 ARPC3 1 3	498,60	-	1,15	0,14	0,75	0,85	-0,16	0,25	0,74	-0,30	0,10
ARPC4_HUMAN	Actin related protein 2 3 complex subunit 4 ARPC4 1 3	2202,81	-	AC	AC	AC	0,69	-0,37	0,06	HAM/TSP	HAM/TSP	HAM/TSP
ARPC5_HUMAN	Actin related protein 2 3 complex subunit 5 ARPC5 1 3	2055,99	AC	AC	AC	AC	AC	AC	AC	-	-	-
ARP2_HUMAN	Actin related protein 2 ACTR2 1 1	669,83	-	AC	AC	AC	0,69	-0,37	0,22	HAM/TSP	HAM/TSP	HAM/TSP
ARP3_HUMAN	Actin related protein 3 ACTR3 1 3	2107,79	-	0,78	-0,25	0,00	0,83	-0,19	0,03	1,05	0,05	0,68
APT_HUMAN	Adenine phosphoribosyltransferase APRT 1 2	657,44	CTR	CTR	CTR	CTR	-	-	-	CTR	CTR	CTR
KAD2_HUMAN	Adenylate kinase 2 mitochondrial AK2 1 2	1162,12	HAM/TSP	-	-	-	HAM/TSP	HAM/TSP	HAM/TSP	HAM/TSP	HAM/TSP	HAM/TSP
CAP1_HUMAN	Adenylyl cyclase associated protein 1 CAP1 1 5	6708,93	-	0,59	-0,52	0,00	1,20	0,18	0,96	2,03	0,71	1,00
ARF1_HUMAN	ADP ribosylation factor 1 ARF1 1 2	2173,73	-	1,67	0,51	0,81	0,89	-0,12	0,44	0,53	-0,63	0,19
ARF3_HUMAN	ADP ribosylation factor 3 ARF3 1 2	457,99	-	CTR	CTR	CTR	HAM/TSP	HAM/TSP	HAM/TSP	0,39	-0,93	0,10
ARF4_HUMAN	ADP ribosylation factor 4 ARF4 1 3	457,99	-	CTR	CTR	CTR	HAM/TSP	HAM/TSP	HAM/TSP	0,93	-0,07	0,44
ARF5_HUMAN	ADP ribosylation factor 5 ARF5 1 2	457,99	-	CTR	CTR	CTR	HAM/TSP	HAM/TSP	HAM/TSP	0,23	-1,47	0,07
ADH1_YEAST	Alcohol dehydrogenase 1 Saccharomyces cerevisiae strain ATCC 204508 S288c ADH1 1 5	8576,68	-	0,53	-0,63	0,00	1,86	0,62	1,00	3,46	1,24	1,00
ACTN1_HUMAN	Alpha actinin 1 ACTN1 1 2	6482,32	-	0,63	-0,46	0,00	1,68	0,52	1,00	2,66	0,98	1,00
ACTN2_HUMAN	Alpha actinin 2 ACTN2 1 1	804,62	-	0,52	-0,65	0,00	1,11	0,10	0,78	2,12	0,75	1,00
ACTN3_HUMAN	Alpha actinin 3 ACTN3 1 2	713,03	-	0,57	-0,56	0,01	0,64	-0,45	0,00	1,12	0,11	0,69

ACTN4_HUMAN	Alpha actinin 4 ACTN4 1 2	2715,76	-	0,67	-0,40	0,00	1,57	0,45	1,00	2,34	0,85	1,00
ENOA_HUMAN	Alpha enolase ENO1 1 2	11032,59	-	1,19	0,17	0,99	1,16	0,15	1,00	0,99	-0,01	0,46
AINX_HUMAN	Alpha internexin INA 1 2	277,58	AC	AC	AC	AC	AC	AC	AC	-	-	-
APC10_HUMAN	Anaphase promoting complex subunit 10 ANAPC10 1 1	376,63	CTR	CTR	CTR	CTR	-	-	-	CTR	CTR	CTR
ANGP2_HUMAN	Angiopoietin 2 ANGPT2 1 1	259,69	AC	AC	AC	AC	AC	AC	AC	-	-	-
ANRA2_HUMAN	Ankyrin repeat family A protein 2 ANKRA2 1 1	292,56	-	1,60	0,47	0,89	AC	AC	AC	CTR	CTR	CTR
ANXA1_HUMAN	Annexin A1 ANXA1 1 2	2832,79	-	1,23	0,21	1,00	1,45	0,37	1,00	1,17	0,16	0,95
ANXA2_HUMAN	Annexin A2 ANXA2 1 2	4543,82	-	1,11	0,10	0,81	1,06	0,06	0,64	0,97	-0,03	0,40
ANXA5_HUMAN	Annexin A5 ANXA5 1 2	5342,11	-	1,12	0,11	0,86	0,60	-0,51	0,00	0,53	-0,63	0,00
ANXA6_HUMAN	Annexin A6 ANXA6 1 3	902,87	-	1,04	0,04	0,55	1,25	0,22	0,93	1,20	0,18	0,84
ATPB_HUMAN	ATP synthase subunit beta mitochondrial ATP5B 1 3	223,49	AC	AC	AC	AC	AC	AC	AC	-	-	-
B2MG_HUMAN	Beta 2 microglobulin B2M 1 1	2103,62	HAM/TSP	-	-	-	HAM/TSP	HAM/TSP	HAM/TSP	HAM/TSP	HAM/TSP	HAM/TSP
ACTBL_HUMAN	Beta actin like protein 2 ACTBL2 1 2	15250,31	-	0,83	-0,19	0,00	0,07	-2,63	0,00	0,09	-2,43	0,00
ENOB_HUMAN	Beta enolase ENO3 1 5	4496,06	-	13,60	2,61	1,00	4,48	1,50	1,00	0,33	-1,11	0,00
SIAT1_HUMAN	Beta galactoside alpha 2 6 sialyltransferase 1 ST6GAL1 1 1	250,53	CTR	CTR	CTR	CTR	-	-	-	CTR	CTR	CTR
BIN2_HUMAN	Bridging integrator 2 BIN2 1 3	310,81	-	AC	AC	AC	1,32	0,28	0,77	HAM/TSP	HAM/TSP	HAM/TSP
KCD12_HUMAN	BTB POZ domain containing protein KCTD12 KCTD12 1 1	470,95	HAM/TSP	-	-	-	HAM/TSP	HAM/TSP	HAM/TSP	HAM/TSP	HAM/TSP	HAM/TSP
KCD16_HUMAN	BTB POZ domain containing protein KCTD16 KCTD16 2 1	288,59	AC	AC	AC	AC	AC	AC	AC	-	-	-
CALD1_HUMAN	Caldesmon CALD1 1 3	962,42	HAM/TSP	-	-	-	HAM/TSP	HAM/TSP	HAM/TSP	HAM/TSP	HAM/TSP	HAM/TSP

CALM_HUMAN	Calmodulin CALM1 1 2	2601,86	HAM/TSP	-	-	-	HAM/TSP	HAM/TSP	HAM/TSP	HAM/TSP	HAM/TSP	HAM/TSP
CALR_HUMAN	Calreticulin CALR 1 1	860,12	-	0,91	-0,09	0,33	1,01	0,01	0,50	1,11	0,10	0,62
CAH2_HUMAN	Carbonic anhydrase 2 CA2 1 2	1027,18	HAM/TSP	-	-	-	HAM/TSP	HAM/TSP	HAM/TSP	HAM/TSP	HAM/TSP	HAM/TSP
CATA_HUMAN	Catalase CAT 1 3	853,28	-	0,71	-0,34	0,04	1,30	0,26	0,95	1,80	0,59	1,00
CAMP_HUMAN	Cathelicidin antimicrobial peptide CAMP 1 1	326,72	HAM/TSP	-	-	-	HAM/TSP	HAM/TSP	HAM/TSP	HAM/TSP	HAM/TSP	HAM/TSP
CATD_HUMAN	Cathepsin D CTSD 1 1	200,39	-	CTR	CTR	CTR	HAM/TSP	HAM/TSP	HAM/TSP	0,51	-0,67	0,19
CDC42_HUMAN	Cell division control protein 42 homolog CDC42 1 2	4433,24	-	1,19	0,17	0,84	1,28	0,25	0,83	1,08	0,08	0,61
CEP19_HUMAN	Centrosomal protein of 19 kDa CEP19 1 2	416,43	CTR	CTR	CTR	CTR	-	-	-	CTR	CTR	CTR
CLIC1_HUMAN	Chloride intracellular channel protein 1 CLIC1 1 4	2680,85	-	0,83	-0,19	0,10	1,25	0,22	0,90	1,52	0,42	1,00
CMA1_HUMAN	Chymase CMA1 1 1	231,40	CTR	CTR	CTR	CTR	-	-	-	CTR	CTR	CTR
COTL1_HUMAN	Coactosin like protein COTL1 1 3	3187,95	-	0,43	-0,84	0,00	1,80	0,59	1,00	4,22	1,44	1,00
F13A_HUMAN	Coagulation factor XIII A chain F13A1 1 4	741,14	-	0,84	-0,18	0,28	0,68	-0,39	0,09	0,81	-0,21	0,22
COF1_HUMAN	Cofilin 1 CFL1 1 3	24039,76	-	1,08	0,08	0,92	1,20	0,18	0,74	1,11	0,10	0,68
COF2_HUMAN	Cofilin 2 CFL2 1 1	3157,37	-	1,13	0,12	0,78	0,39	-0,95	0,00	0,35	-1,06	0,00
H2AY_HUMAN	Core histone macro H2A 1 H2AFY 1 4	335,54	AC	AC	AC	AC	AC	AC	AC	-	-	-
COR1A_HUMAN	Coronin 1A CORO1A 1 4	5506,56	-	0,91	-0,09	0,45	1,54	0,43	1,00	1,67	0,51	0,82
COR1C_HUMAN	Coronin 1C CORO1C 1 1	501,00	-	-	-	-	1,92	0,65	0,99	HAM/TSP	HAM/TSP	HAM/TSP
CRKL_HUMAN	Crk like protein CRKL 1 1	652,25	HAM/TSP	-	-	-	HAM/TSP	HAM/TSP	HAM/TSP	HAM/TSP	HAM/TSP	HAM/TSP
CYTB_HUMAN	Cystatin B CSTB 1 2	1610,54	-	CTR	CTR	CTR	HAM/TSP	HAM/TSP	HAM/TSP	1,14	0,13	0,64
CSRP1_HUMAN	Cysteine and glycine rich protein 1 CSRP1 1 3	308,31	HAM/TSP	-	-	-	HAM/TSP	HAM/TSP	HAM/TSP	HAM/TSP	HAM/TSP	HAM/TSP
CYC_HUMAN	Cytochrome c CYCS 1 2	545,16	HAM/TSP	-	-	-	HAM/TSP	HAM/TSP	HAM/TSP	HAM/TSP	HAM/TSP	HAM/TSP
DESM_HUMAN	Desmin DES 1 3	256,88	AC	AC	AC	AC	AC	AC	AC	-	-	-
DEST_HUMAN	Destrin DSTN 1 3	311,33	HAM/TSP	-	-	-	HAM/TSP	HAM/TSP	HAM/TSP	HAM/TSP	HAM/TSP	HAM/TSP

DPYL2_HUMAN	Dihydropyrimidinase related protein 2 DPYSL2 1 1	223,56	AC	AC	AC	AC	AC	AC	AC	-	-	-
EF1A1_HUMAN	Elongation factor 1 alpha 1 EEF1A1 1 1	880,38	-	1,49	0,40	1,00	1,38	0,32	0,90	0,92	-0,08	0,32
EF1A2_HUMAN	Elongation factor 1 alpha 2 EEF1A2 1 1	563,58	-	1,80	0,59	1,00	1,52	0,42	0,89	0,85	-0,16	0,30
EF1G_HUMAN	Elongation factor 1 gamma EEF1G 1 3	572,77	HAM/TSP	-	-	-	HAM/TSP	HAM/TSP	HAM/TSP	HAM/TSP	HAM/TSP	HAM/TSP
EF2_HUMAN	Elongation factor 2 EEF2 1 4	272,13	-	1,09	0,09	0,54	0,87	-0,14	0,40	0,79	-0,23	0,40
ERP29_HUMAN	Endoplasmic reticulum resident protein 29 ERP29 1 4	1245,99	HAM/TSP	-	-	-	HAM/TSP	HAM/TSP	HAM/TSP	HAM/TSP	HAM/TSP	HAM/TSP
ENPL_HUMAN	Endoplasmin HSP90B1 1 1	679,66	HAM/TSP	-	-	-	HAM/TSP	HAM/TSP	HAM/TSP	HAM/TSP	HAM/TSP	HAM/TSP
LPPL_HUMAN	Eosinophil lysophospholipase CLC 1 3	1473,99	HAM/TSP	-	-	-	HAM/TSP	HAM/TSP	HAM/TSP	HAM/TSP	HAM/TSP	HAM/TSP
PERE_HUMAN	Eosinophil peroxidase EPX 1 2	355,57	AC	AC	AC	AC	AC	AC	AC	-	-	-
IF5A1_HUMAN	Eukaryotic translation initiation factor 5A 1 EIF5A 1 2	1702,23	HAM/TSP	-	-	-	HAM/TSP	HAM/TSP	HAM/TSP	HAM/TSP	HAM/TSP	HAM/TSP
IF5AL_HUMAN	Eukaryotic translation initiation factor 5A 1 like EIF5AL1 1 2	1542,83	HAM/TSP	-	-	-	HAM/TSP	HAM/TSP	HAM/TSP	HAM/TSP	HAM/TSP	HAM/TSP
IF5A2_HUMAN	Eukaryotic translation initiation factor 5A 2 EIF5A2 1 3	2828,50	HAM/TSP	-	-	-	HAM/TSP	HAM/TSP	HAM/TSP	HAM/TSP	HAM/TSP	HAM/TSP
EZRI_HUMAN	Ezrin EZR 1 4	563,14	-	1,16	0,15	0,93	0,95	-0,05	0,37	0,82	-0,20	0,03
CAZA1_HUMAN	F actin capping protein subunit alpha 1 CAPZA1 1 3	3251,20	-	0,78	-0,25	0,05	0,84	-0,18	0,08	1,06	0,06	0,67
CAZA2_HUMAN	F actin capping protein subunit alpha 2 CAPZA2 1 3	1252,32	HAM/TSP	-	-	-	HAM/TSP	HAM/TSP	HAM/TSP	HAM/TSP	HAM/TSP	HAM/TSP
CAPZB_HUMAN	F actin capping protein subunit beta CAPZB 1 4	1646,12	-	AC	AC	AC	0,78	-0,25	0,19	HAM/TSP	HAM/TSP	HAM/TSP

URP2_HUMAN	Fermitin family homolog 3 FERMT3 1 1	857,98	-	0,61	-0,49	0,01	1,15	0,14	0,81	1,88	0,63	1,00
FEZF2_HUMAN	Fez family zinc finger protein 2 FEZF2 2 2	250,39	HAM/TSP	-	-	-	HAM/TSP	HAM/TSP	HAM/TSP	HAM/TSP	HAM/TSP	HAM/TSP
FGOP2_HUMAN	FGFR1 oncogene partner 2 FGFR1OP2 2 1	361,54	HAM/TSP	-	-	-	HAM/TSP	HAM/TSP	HAM/TSP	HAM/TSP	HAM/TSP	HAM/TSP
FIBA_HUMAN	Fibrinogen alpha chain FGA 1 2	1397,74	-	1,06	0,06	0,67	1,52	0,42	1,00	1,43	0,36	1,00
FIBB_HUMAN	Fibrinogen beta chain FGB 1 2	3240,36	-	0,83	-0,19	0,08	1,49	0,40	1,00	1,79	0,58	1,00
FIBG_HUMAN	Fibrinogen gamma chain FGG 1 3	6128,56	-	-	-	-	-	-	-	-	-	-
FGF4_HUMAN	Fibroblast growth factor 4 FGF4 1 1	455,68	CTR	CTR	CTR	CTR	-	-	-	CTR	CTR	CTR
FLNA_HUMAN	Filamin A FLNA 1 4	6991,47	-	0,63	-0,47	0,00	1,62	0,48	1,00	2,59	0,95	1,00
FLNC_HUMAN	Filamin C FLNC 1 3	358,54	HAM/TSP	-	-	-	HAM/TSP	HAM/TSP	HAM/TSP	HAM/TSP	HAM/TSP	HAM/TSP
BLVRB_HUMAN	Flavin reductase NADPH BLVRB 1 3	627,25	AC	AC	AC	AC	AC	AC	AC	-	-	-
F16P1_HUMAN	Fructose 1 6 bisphosphatase 1 FBP1 1 5	421,99	-	1,36	0,31	0,72	AC	AC	AC	CTR	CTR	CTR
ALDOA_HUMAN	Fructose bisphosphate aldolase A ALDOA 1 2	6724,85	-	1,28	0,25	1,00	1,01	0,01	0,53	0,79	-0,24	0,01
ALDOC_HUMAN	Fructose bisphosphate aldolase C ALDOC 1 2	334,92	-	AC	AC	AC	1,16	0,15	0,68	HAM/TSP	HAM/TSP	HAM/TSP
LEG1_HUMAN	Galectin 1 LGALS1 1 2	1649,58	-	1,15	0,14	0,73	0,77	-0,26	0,16	0,67	-0,40	0,09
LEGL_HUMAN	Galectin related protein LGALSL 1 2	2575,04	-	0,78	-0,25	0,18	0,77	-0,26	0,20	0,99	-0,01	0,49
ENOG_HUMAN	Gamma enolase ENO2 1 3	5441,02	-	15,49	2,74	1,00	5,70	1,74	1,00	0,36	-1,01	0,00
GGACT_HUMAN	Gamma glutamylaminecyclotransfe rase GGACT 1 2	309,21	AC	AC	AC	AC	AC	AC	AC	-	-	-
GELS_HUMAN	Gelsolin GSN 1 1	2848,47	-	0,62	-0,48	0,00	2,27	0,82	1,00	3,67	1,30	1,00
GMFG_HUMAN	Glia maturation factor gamma GMFG 1 1	969,51	HAM/TSP	-	-	-	HAM/TSP	HAM/TSP	HAM/TSP	HAM/TSP	HAM/TSP	HAM/TSP
G6PD_HUMAN	Glucose 6 phosphate 1 dehydrogenase G6PD 1 4	551,90	-	1,55	0,44	0,82	0,95	-0,05	0,45	0,61	-0,50	0,07
G6PI_HUMAN	Glucose 6 phosphate isomerase GPI 1 4	744,66	-	1,21	0,19	0,84	1,31	0,27	0,92	1,08	0,08	0,69

GPX1_HUMAN	Glutathione peroxidase 1 GPX1 1 4	657,36	-	AC	AC	AC	0,92	-0,08	0,44	HAM/TSP	HAM/TSP	HAM/TSP
GSTO1_HUMAN	Glutathione S transferase omega 1 GSTO1 1 2	2486,39	HAM/TSP	-	-	-	HAM/TSP	HAM/TSP	HAM/TSP	HAM/TSP	HAM/TSP	HAM/TSP
GSTP1_HUMAN	Glutathione S transferase P GSTP1 1 2	8566,91	-	1,19	0,17	0,99	0,98	-0,02	0,49	0,84	-0,18	0,04
G3P_HUMAN	Glyceraldehyde 3 phosphate dehydrogenase GAPDH 1 3	5039,56	-	1,15	0,14	0,95	1,35	0,30	1,00	1,17	0,16	0,98
GOSR1_HUMAN	Golgi SNAP receptor complex member 1 GOSR1 1 1	283,03	AC	AC	AC	AC	AC	AC	AC	-	-	-
FRAT2_HUMAN	GSK 3 binding protein FRAT2 FRAT2 2 3	249,57	AC	AC	AC	AC	AC	AC	AC	-	-	-
RAN_HUMAN	GTP binding nuclear protein Ran RAN 1 3	2952,08	-	0,70	-0,35	0,20	0,50	-0,70	0,01	0,70	-0,35	0,17
HS71L_HUMAN	Heat shock 70 kDa protein 1 like HSPA1L 1 2	1734,72	-	0,83	-0,19	0,18	0,55	-0,60	0,00	0,66	-0,41	0,00
HSP71_HUMAN	Heat shock 70 kDa protein 1A 1B HSPA1A 1 5	2641,23	-	0,83	-0,19	0,06	0,62	-0,48	0,00	0,75	-0,29	0,00
HSP74_HUMAN	Heat shock 70 kDa protein 4 HSPA4 1 4	310,72	CTR	CTR	CTR	CTR	-	-	-	CTR	CTR	CTR
HSP76_HUMAN	Heat shock 70 kDa protein 6 HSPA6 1 2	1687,35	-	0,79	-0,23	0,14	0,61	-0,50	0,00	0,76	-0,27	0,19
HSP7C_HUMAN	Heat shock cognate 71 kDa protein HSPA8 1 1	4140,69	-	0,70	-0,36	0,00	0,64	-0,44	0,00	0,92	-0,08	0,21
TRAP1_HUMAN	Heat shock protein 75 kDa mitochondrial TRAP1 1 3	769,74	HAM/TSP	-	-	-	HAM/TSP	HAM/TSP	HAM/TSP	HAM/TSP	HAM/TSP	HAM/TSP
HS90A_HUMAN	Heat shock protein HSP 90 alpha HSP90AA1 1 5	439,66	-	-	-	-	1,45	0,37	0,85	HAM/TSP	HAM/TSP	HAM/TSP
HS90B_HUMAN	Heat shock protein HSP 90 beta HSP90AB1 1 4	346,06	-	-	-	-	1,28	0,25	0,75	HAM/TSP	HAM/TSP	HAM/TSP
HSP72_HUMAN	Heat shock related 70 kDa protein 2 HSPA2 1 1	1521,65	-	0,87	-0,14	0,21	2,14	0,76	1,00	2,46	0,90	1,00
HEBP2_HUMAN	Heme binding protein 2 HEBP2 1 1	1035,64	-	CTR	CTR	CTR	HAM/TSP	HAM/TSP	HAM/TSP	0,75	-0,29	0,38
HBA_HUMAN	Hemoglobin subunit alpha HBA1 1 2	987,48	HAM/TSP	-	-	-	HAM/TSP	HAM/TSP	HAM/TSP	HAM/TSP	HAM/TSP	HAM/TSP

HBB_HUMAN	Hemoglobin subunit beta HBB 1 2	1689,48	HAM/TSP	-	-	-	HAM/TSP	HAM/TSP	HAM/TSP	HAM/TSP	HAM/TSP	HAM/TSP
HBD_HUMAN	Hemoglobin subunit delta HBD 1 2	964,89	HAM/TSP	-	-	-	HAM/TSP	HAM/TSP	HAM/TSP	HAM/TSP	HAM/TSP	HAM/TSP
ROA1_HUMAN	Heterogeneous nuclear ribonucleoprotein A1 HNRNPA1 1 5	247,82	HAM/TSP	-	-	-	HAM/TSP	HAM/TSP	HAM/TSP	HAM/TSP	HAM/TSP	HAM/TSP
HNRDL_HUMAN	Heterogeneous nuclear ribonucleoprotein D like HNRPDL 1 3	464,03	AC	AC	AC	AC	AC	AC	AC	-	-	-
HNRPD_HUMAN	Heterogeneous nuclear ribonucleoprotein D0 HNRNPD 1 1	887,02	-	1,07	0,07	0,57	0,35	-1,04	0,01	0,33	-1,11	0,01
HNRPF_HUMAN	Heterogeneous nuclear ribonucleoprotein F HNRNPF 1 3	2096,44	-	1,32	0,28	0,92	0,66	-0,41	0,08	0,50	-0,69	0,00
HNRH1_HUMAN	Heterogeneous nuclear ribonucleoprotein H HNRNPH1 1 4	2149,50	-	1,38	0,32	0,89	0,71	-0,34	0,10	0,51	-0,67	0,01
HNRH2_HUMAN	Heterogeneous nuclear ribonucleoprotein H2 HNRNPH2 1 1	303,15	-	AC	AC	AC	0,84	-0,18	0,33	HAM/TSP	HAM/TSP	HAM/TSP
HNRPQ_HUMAN	Heterogeneous nuclear ribonucleoprotein Q SYNCRIP 1 2	403,76	-	AC	AC	AC	0,56	-0,58	0,16	HAM/TSP	HAM/TSP	HAM/TSP
HNRPR_HUMAN	Heterogeneous nuclear ribonucleoprotein R HNRNPR 1 1	386,30	AC	AC	AC	AC	AC	AC	AC	-	-	-
ROA2_HUMAN	Heterogeneous nuclear ribonucleoproteins A2 B1 HNRNPA2B1 1 2	1844,49	-	0,90	-0,10	0,36	0,41	-0,88	0,00	0,46	-0,78	0,02
HMGB1_HUMAN	High mobility group protein B1 HMGB1 1 3	1752,90	HAM/TSP	-	-	-	HAM/TSP	HAM/TSP	HAM/TSP	HAM/TSP	HAM/TSP	HAM/TSP
HMGB2_HUMAN	High mobility group protein B2 HMGB2 1 2	857,12	-	AC	AC	AC	1,22	0,20	0,75	HAM/TSP	HAM/TSP	HAM/TSP
H11_HUMAN	Histone H1 1 HIST1H1A 1 3	504,28	CTR	CTR	CTR	CTR	-	-	-	CTR	CTR	CTR
H12_HUMAN	Histone H1 2 HIST1H1C 1 2	1740,51	-	3,16	1,15	1,00	0,37	-0,99	0,00	0,12	-2,14	0,00
H13_HUMAN	Histone H1 3 HIST1H1D 1 2	1153,04	-	0,58	-0,55	0,01	0,48	-0,73	0,01	0,84	-0,17	0,32
H14_HUMAN	Histone H1 4 HIST1H1E 1 2	1153,04	-	0,55	-0,59	0,01	0,53	-0,63	0,01	0,96	-0,04	0,51

H15_HUMAN	Histone H1 5 HIST1H1B 1 3	605,82	-	1,75	0,56	0,89	AC	AC	AC	CTR	CTR	CTR
H1T_HUMAN	Histone H1t HIST1H1T 2 4	504,28	CTR	CTR	CTR	CTR	-	-	-	CTR	CTR	CTR
H2AJ_HUMAN	Histone H2A J H2AFJ 1 1	8816,08	-	1,99	0,69	1,00	0,13	-2,02	0,00	0,07	-2,71	0,00
H2A1A_HUMAN	Histone H2A type 1 A HIST1H2AA 1 3	8216,94	-	2,12	0,75	1,00	0,41	-0,90	0,00	0,19	-1,65	0,00
H2A1B_HUMAN	Histone H2A type 1 B E HIST1H2AB 1 2	8816,08	-	2,03	0,71	1,00	0,14	-1,97	0,00	0,07	-2,67	0,00
H2A1C_HUMAN	Histone H2A type 1 C HIST1H2AC 1 3	8816,08	-	1,99	0,69	1,00	0,13	-2,03	0,00	0,07	-2,72	0,00
H2A1D_HUMAN	Histone H2A type 1 D HIST1H2AD 1 2	10150,99	-	2,01	0,70	1,00	0,13	-2,06	0,00	0,06	-2,75	0,00
H2A1H_HUMAN	Histone H2A type 1 H HIST1H2AH 1 3	8216,94	-	2,05	0,72	1,00	0,49	-0,72	0,00	0,24	-1,44	0,00
H2A1_HUMAN	Histone H2A type 1 HIST1H2AG 1 2	8816,08	-	2,01	0,70	1,00	0,14	-1,98	0,00	0,07	-2,69	0,00
H2A1J_HUMAN	Histone H2A type 1 J HIST1H2AJ 1 3	8816,08	-	2,01	0,70	1,00	0,14	-2,00	0,00	0,07	-2,70	0,00
H2A2A_HUMAN	Histone H2A type 2 A HIST2H2AA3 1 3	8816,08	-	2,03	0,71	1,00	0,14	-1,96	0,00	0,07	-2,67	0,00
H2A2B_HUMAN	Histone H2A type 2 B HIST2H2AB 1 3	7665,42	-	2,14	0,76	1,00	AC	AC	AC	CTR	CTR	CTR
H2A2C_HUMAN	Histone H2A type 2 C HIST2H2AC 1 4	8816,08	-	2,03	0,71	1,00	0,14	-1,96	0,00	0,07	-2,67	0,00
H2A3_HUMAN	Histone H2A type 3 HIST3H2A 1 3	8816,08	-	1,99	0,69	1,00	0,13	-2,03	0,00	0,07	-2,72	0,00
H2AV_HUMAN	Histone H2A V H2AFV 1 3	3260,50	-	1,92	0,65	1,00	0,10	-2,35	0,00	0,05	-3,00	0,00
H2AX_HUMAN	Histone H2A x H2AFX 1 2	8216,94	-	2,10	0,74	1,00	0,35	-1,06	0,00	0,17	-1,80	0,00
H2AZ_HUMAN	Histone H2A Z H2AFZ 1 2	3249,28	-	1,92	0,65	1,00	0,09	-2,44	0,00	0,05	-3,09	0,00
H2B1A_HUMAN	Histone H2B type 1 A HIST1H2BA 1 3	8742,52	-	1,36	0,31	1,00	0,39	-0,93	0,23	0,29	-1,24	0,08
H2B1B_HUMAN	Histone H2B type 1 B HIST1H2BB 1 2	11374,98	-	1,55	0,44	1,00	0,14	-1,97	0,00	0,09	-2,41	0,00
H2B1C_HUMAN	Histone H2B type 1 C E F G I HIST1H2BC 1 4	12877,73	-	1,45	0,37	1,00	0,19	-1,65	0,00	0,13	-2,01	0,00
H2B1D_HUMAN	Histone H2B type 1 D HIST1H2BD 1 2	13156,22	-	1,39	0,33	1,00	0,19	-1,67	0,00	0,14	-1,99	0,00
H2B1H_HUMAN	Histone H2B type 1 H HIST1H2BH 1 3	13156,22	-	1,42	0,35	1,00	0,20	-1,62	0,00	0,14	-1,97	0,00

H2B1J_HUMAN	Histone H2B type 1 J HIST1H2BJ 1 3	11374,98	-	1,54	0,43	1,00	0,14	-1,96	0,00	0,09	-2,39	0,00
H2B1K_HUMAN	Histone H2B type 1 K HIST1H2BK 1 3	13156,22	-	1,36	0,31	0,99	0,18	-1,70	0,00	0,13	-2,01	0,00
H2B1L_HUMAN	Histone H2B type 1 L HIST1H2BL 1 3	13156,22	-	1,40	0,34	1,00	0,19	-1,68	0,00	0,13	-2,02	0,00
H2B1M_HUMAN	Histone H2B type 1 M HIST1H2BM 1 3	13156,22	-	1,39	0,33	1,00	0,19	-1,66	0,00	0,14	-1,99	0,00
H2B1N_HUMAN	Histone H2B type 1 N HIST1H2BN 1 3	13156,22	-	1,40	0,34	1,00	0,18	-1,72	0,00	0,13	-2,06	0,00
H2B1O_HUMAN	Histone H2B type 1 O HIST1H2BO 1 3	11501,00	-	1,54	0,43	1,00	0,14	-1,97	0,00	0,09	-2,40	0,00
H2B2E_HUMAN	Histone H2B type 2 E HIST2H2BE 1 3	11374,98	-	1,55	0,44	1,00	0,15	-1,92	0,00	0,09	-2,36	0,00
H2B2F_HUMAN	Histone H2B type 2 F HIST2H2BF 1 3	18964,46	-	1,35	0,30	1,00	0,17	-1,75	0,00	0,13	-2,05	0,00
H2B3B_HUMAN	Histone H2B type 3 B HIST3H2BB 1 3	10387,22	-	1,52	0,42	1,00	0,19	-1,68	0,00	0,12	-2,10	0,00
H2BFS_HUMAN	Histone H2B type F S H2BFS 1 2	13156,22	-	1,40	0,34	1,00	0,19	-1,66	0,00	0,14	-2,00	0,00
H31_HUMAN	Histone H3 1 HIST1H3A 1 2	1858,10	-	1,68	0,52	0,89	1,77	0,57	0,74	1,04	0,04	0,57
H31T_HUMAN	Histone H3 1t HIST3H3 1 3	2819,27	-	1,04	0,04	0,58	0,75	-0,29	0,15	0,72	-0,33	0,18
H32_HUMAN	Histone H3 2 HIST2H3A 1 3	1858,10	-	1,67	0,51	0,89	1,30	0,26	0,57	0,78	-0,25	0,40
H33_HUMAN	Histone H3 3 H3F3A 1 2	1858,10	-	1,28	0,25	0,69	1,14	0,13	0,48	0,89	-0,12	0,42
H3C_HUMAN	Histone H3 3C H3F3C 1 3	1858,10	-	1,26	0,23	0,67	1,49	0,40	0,75	1,19	0,17	0,71
H4_HUMAN	Histone H4 HIST1H4A 1 2	21460,10	-	1,46	0,38	1,00	0,39	-0,93	0,00	0,27	-1,31	0,00
IGHG1_HUMAN	Ig gamma 1 chain C region IGHG1 1 1	1246,10	-	0,53	-0,63	0,00	0,84	-0,17	0,23	1,60	0,47	0,95
IGHG2_HUMAN	Ig gamma 2 chain C region IGHG2 1 2	639,92	-	-	-	-	0,58	-0,54	0,08	HAM/TSP	HAM/TSP	HAM/TSP
IGHG3_HUMAN	Ig gamma 3 chain C region IGHG3 1 2	815,88	-	AC	AC	AC	0,55	-0,60	0,05	HAM/TSP	HAM/TSP	HAM/TSP
IGHG4_HUMAN	Ig gamma 4 chain C region IGHG4 1 1	557,59	-	-	-	-	0,69	-0,37	0,14	HAM/TSP	HAM/TSP	HAM/TSP
IGKC_HUMAN	Ig kappa chain C region IGKC 1 1	2861,64	-	0,74	-0,30	0,16	1,19	0,17	0,75	1,60	0,47	0,94
LAC1_HUMAN	Ig lambda 1 chain C regions IGLC1 1 1	1550,19	HAM/TSP	-	-	-	HAM/TSP	HAM/TSP	HAM/TSP	HAM/TSP	HAM/TSP	HAM/TSP

LAC2_HUMAN	Ig lambda 2 chain C regions IGLC2 1 1	1592,26	HAM/TSP	-	-	-	HAM/TSP	HAM/TSP	HAM/TSP	HAM/TSP	HAM/TSP	HAM/TSP
LAC3_HUMAN	Ig lambda 3 chain C regions IGLC3 1 1	1592,26	HAM/TSP	-	-	-	HAM/TSP	HAM/TSP	HAM/TSP	HAM/TSP	HAM/TSP	HAM/TSP
LAC6_HUMAN	Ig lambda 6 chain C region IGLC6 4 1	1559,30	HAM/TSP	-	-	-	HAM/TSP	HAM/TSP	HAM/TSP	HAM/TSP	HAM/TSP	HAM/TSP
LAC7_HUMAN	Ig lambda 7 chain C region IGLC7 1 2	1078,13	HAM/TSP	-	-	-	HAM/TSP	HAM/TSP	HAM/TSP	HAM/TSP	HAM/TSP	HAM/TSP
IGLL5_HUMAN	Immunoglobulin lambda like polypeptide 5 IGLL5 2 2	1592,26	HAM/TSP	-	-	-	HAM/TSP	HAM/TSP	HAM/TSP	HAM/TSP	HAM/TSP	HAM/TSP
IMB1_HUMAN	Importin subunit beta 1 KPNB1 1 2	408,74	HAM/TSP	-	-	-	HAM/TSP	HAM/TSP	HAM/TSP	HAM/TSP	HAM/TSP	HAM/TSP
ICEF1_HUMAN	Interactor protein for cytohesin exchange factors 1 IPCEF1 1 1	328,23	AC	AC	AC	AC	AC	AC	AC	-	-	-
IL6RA_HUMAN	Interleukin 6 receptor subunit alpha IL6R 1 1	386,06	HAM/TSP	-	-	-	HAM/TSP	HAM/TSP	HAM/TSP	HAM/TSP	HAM/TSP	HAM/TSP
IDHC_HUMAN	Isocitrate dehydrogenase NADP cytoplasmic IDH1 1 2	262,01	CTR	CTR	CTR	CTR	-	-	-	CTR	CTR	CTR
JOS2_HUMAN	Josephin 2 JOSD2 1 1	169,33	CTR	CTR	CTR	CTR	-	-	-	CTR	CTR	CTR
KIF22_HUMAN	Kinesin like protein KIF22 KIF22 1 5	221,12	HAM/TSP	-	-	-	HAM/TSP	HAM/TSP	HAM/TSP	HAM/TSP	HAM/TSP	HAM/TSP
LDHA_HUMAN	L lactate dehydrogenase A chain LDHA 1 2	323,82	CTR	CTR	CTR	CTR	-	-	-	CTR	CTR	CTR
LDHB_HUMAN	L lactate dehydrogenase B chain LDHB 1 2	2475,68	-	0,83	-0,19	0,20	0,68	-0,39	0,04	0,82	-0,20	0,23
LASP1_HUMAN	LIM and SH3 domain protein 1 LASP1 1 2	288,26	-	AC	AC	AC	1,26	0,23	0,63	HAM/TSP	HAM/TSP	HAM/TSP
LSP1_HUMAN	Lymphocyte specific protein 1 LSP1 1 1	370,38	-	1,43	0,36	0,82	AC	AC	AC	CTR	CTR	CTR
LYSC_HUMAN	Lysozyme C LYZ 1 1	36511,90	-	1,20	0,18	1,00	0,79	-0,24	0,00	0,66	-0,42	0,00
NIPA3_HUMAN	Magnesium transporter NIPA3 NIPAL1 2 1	231,98	HAM/TSP	-	-	-	HAM/TSP	HAM/TSP	HAM/TSP	HAM/TSP	HAM/TSP	HAM/TSP
MDHC_HUMAN	Malate dehydrogenase cytoplasmic MDH1 1 4	1015,27	CTR	CTR	CTR	CTR	-	-	-	CTR	CTR	CTR
MDHM_HUMAN	Malate dehydrogenase mitochondrial MDH2 1 3	3995,38	-	1,23	0,21	0,82	0,71	-0,34	0,08	0,58	-0,55	0,01

MANF_HUMAN	Mesencephalic astrocyte derived neurotrophic factor MANF 1 3	1067,99	HAM/TSP	-	-	-	HAM/TSP	HAM/TSP	HAM/TSP	HAM/TSP	HAM/TSP	HAM/TSP
GRM6_HUMAN	Metabotropic glutamate receptor 6 GRM6 1 2	253,50	CTR	CTR	CTR	CTR	-	-	-	CTR	CTR	CTR
MARE1_HUMAN	Microtubule associated protein RP EB family member 1 MAPRE1 1 3	601,13	HAM/TSP	-	-	-	HAM/TSP	HAM/TSP	HAM/TSP	HAM/TSP	HAM/TSP	HAM/TSP
MOES_HUMAN	Moesin MSN 1 3	3280,52	-	1,32	0,28	1,00	1,19	0,17	0,98	0,90	-0,11	0,13
MNDA_HUMAN	Myeloid cell nuclear differentiation antigen MNDA 1 1	2391,33	-	0,84	-0,17	0,15	0,76	-0,27	0,05	0,90	-0,10	0,27
PERM_HUMAN	Myeloperoxidase MPO 1 1	5127,57	-	1,36	0,31	1,00	0,61	-0,50	0,00	0,45	-0,80	0,00
MYH9_HUMAN	Myosin 9 MYH9 1 4	228,08	-	0,73	-0,31	0,05	0,85	-0,16	0,13	1,16	0,15	0,80
MYL6B_HUMAN	Myosin light chain 6B MYL6B 1 1	710,45	-	1,77	0,57	1,00	1,25	0,22	0,67	0,70	-0,36	0,19
MYL6_HUMAN	Myosin light polypeptide 6 MYL6 1 2	2120,76	-	0,96	-0,04	0,44	1,12	0,11	0,71	1,17	0,16	0,81
ML12A_HUMAN	Myosin regulatory light chain 12A MYL12A 1 2	458,51	HAM/TSP	-	-	-	HAM/TSP	HAM/TSP	HAM/TSP	HAM/TSP	HAM/TSP	HAM/TSP
ML12B_HUMAN	Myosin regulatory light chain 12B MYL12B 1 2	448,68	-	CTR	CTR	CTR	-	-	-	1,93	0,66	0,74
MYL9_HUMAN	Myosin regulatory light polypeptide 9 MYL9 1 4	293,06	HAM/TSP	-	-	-	HAM/TSP	HAM/TSP	HAM/TSP	HAM/TSP	HAM/TSP	HAM/TSP
MTPN_HUMAN	Myotrophin MTPN 1 2	2669,06	HAM/TSP	-	-	-	HAM/TSP	HAM/TSP	HAM/TSP	HAM/TSP	HAM/TSP	HAM/TSP
NCF2_HUMAN	Neutrophil cytosol factor 2 NCF2 1 2	471,45	HAM/TSP	-	-	-	HAM/TSP	HAM/TSP	HAM/TSP	HAM/TSP	HAM/TSP	HAM/TSP
ELNE_HUMAN	Neutrophil elastase ELANE 1 1	787,99	-	AC	AC	AC	0,55	-0,59	0,19	-	-	-
RNAS2_HUMAN	Non secretory ribonuclease RNASE2 1 2	4772,35	HAM/TSP	-	-	-	HAM/TSP	HAM/TSP	HAM/TSP	HAM/TSP	HAM/TSP	HAM/TSP
NPM_HUMAN	Nucleophosmin NPM1 1 2	565,26	-	1,22	0,20	0,64	0,48	-0,73	0,06	0,40	-0,92	0,00
NDKA_HUMAN	Nucleoside diphosphate kinase A NME1 1 1	950,49	-	AC	AC	AC	1,62	0,48	0,93	HAM/TSP	HAM/TSP	HAM/TSP
NDKB_HUMAN	Nucleoside diphosphate kinase B NME2 1 1	1436,94	-	AC	AC	AC	1,55	0,44	0,96	HAM/TSP	HAM/TSP	HAM/TSP
OSTF1_HUMAN	Osteoclast stimulating factor 1 OSTF1 1 2	635,27	HAM/TSP	-	-	-	HAM/TSP	HAM/TSP	HAM/TSP	HAM/TSP	HAM/TSP	HAM/TSP

PDLI1_HUMAN	PDZ and LIM domain protein 1 PDLIM1 1 4	1395,51	-	AC	AC	AC	1,92	0,65	1,00	HAM/TSP	HAM/TSP	HAM/TSP
PPIA_HUMAN	Peptidyl prolyl cis trans isomerase A PPIA 1 2	2960,08	-	1,07	0,07	0,66	1,58	0,46	1,00	1,48	0,39	0,99
PAL4A_HUMAN	Peptidyl prolyl cis trans isomerase A like 4A B C PPIAL4A 2 1	1731,81	-	1,11	0,10	0,60	0,89	-0,12	0,36	0,80	-0,22	0,23
PPIB_HUMAN	Peptidyl prolyl cis trans isomerase B PPIB 1 2	1683,35	-	0,79	-0,23	0,18	0,79	-0,24	0,10	0,99	-0,01	0,44
FKB1A_HUMAN	Peptidyl prolyl cis trans isomerase FKBP1A FKBP1A 1 2	4605,23	HAM/TSP	-	-	-	HAM/TSP	HAM/TSP	HAM/TSP	HAM/TSP	HAM/TSP	HAM/TSP
PRDX1_HUMAN	Peroxiredoxin 1 PRDX1 1 1	1236,46	-	0,61	-0,50	0,05	0,73	-0,31	0,17	1,21	0,19	0,67
PRDX2_HUMAN	Peroxiredoxin 2 PRDX2 1 5	437,53	HAM/TSP	-	-	-	HAM/TSP	HAM/TSP	HAM/TSP	HAM/TSP	HAM/TSP	HAM/TSP
PRDX4_HUMAN	Peroxiredoxin 4 PRDX4 1 1	451,76	AC	AC	AC	AC	AC	AC	AC	-	-	-
PRDX5_HUMAN	Peroxiredoxin 5 mitochondrial PRDX5 1 4	1583,95	-	0,97	-0,03	0,44	0,76	-0,27	0,16	0,79	-0,24	0,24
PRDX6_HUMAN	Peroxiredoxin 6 PRDX6 1 3	2549,12	-	AC	AC	AC	0,50	-0,69	0,00	HAM/TSP	HAM/TSP	HAM/TSP
PEBP1_HUMAN	Phosphatidylethanolamine binding protein 1 PEBP1 1 3	2745,21	HAM/TSP	-	-	-	HAM/TSP	HAM/TSP	HAM/TSP	HAM/TSP	HAM/TSP	HAM/TSP
PI42A_HUMAN	Phosphatidylinositol 5 phosphate 4 kinase type 2 alpha PIP4K2A 1 2	303,88	HAM/TSP	-	-	-	HAM/TSP	HAM/TSP	HAM/TSP	HAM/TSP	HAM/TSP	HAM/TSP
PGK1_HUMAN	Phosphoglycerate kinase 1 PGK1 1 3	6226,27	-	1,12	0,11	0,86	1,07	0,07	0,76	0,97	-0,03	0,33
PGK2_HUMAN	Phosphoglycerate kinase 2 PGK2 1 3	783,52	-	0,87	-0,14	0,32	1,34	0,29	0,97	1,52	0,42	0,94
PGAM1_HUMAN	Phosphoglycerate mutase 1 PGAM1 1 2	3905,02	-	1,05	0,05	0,64	1,12	0,11	0,79	1,06	0,06	0,67
PGAM2_HUMAN	Phosphoglycerate mutase 2 PGAM2 1 3	2913,20	-	1,07	0,07	0,67	0,88	-0,13	0,20	0,82	-0,20	0,09
PLSL_HUMAN	Plastin 2 LCP1 1 6	4479,28	-	1,09	0,09	0,89	1,09	0,09	0,84	1,00	0,00	0,50
PLST_HUMAN	Plastin 3 PLS3 1 4	468,81	-	0,66	-0,41	0,07	0,83	-0,19	0,24	1,25	0,22	0,79
CXCL7_HUMAN	Platelet basic protein PPBP 1 3	36112,73	-	0,90	-0,11	0,00	1,42	0,35	1,00	1,58	0,46	1,00
PLF4_HUMAN	Platelet factor 4 PF4 1 2	21220,50	-	1,03	0,03	0,67	2,64	0,97	1,00	2,59	0,95	1,00

PF4V_HUMAN	Platelet factor 4 variant PF4V1 1 1	11943,67	-	1,00	0,00	0,47	2,89	1,06	1,00	2,92	1,07	1,00
PLEK_HUMAN	Pleckstrin PLEK 1 3	3843,49	-	0,66	-0,42	0,00	1,08	0,08	0,78	1,67	0,51	1,00
PKHF1_HUMAN	Pleckstrin homology domain containing family F member 1 PLEKHF1 2 3	352,49	HAM/TSP	-	-	-	HAM/TSP	HAM/TSP	HAM/TSP	HAM/TSP	HAM/TSP	HAM/TSP
UBB_HUMAN	Polyubiquitin B UBB 1 1	2033,26	-	1,38	0,32	0,76	3,25	1,18	1,00	2,39	0,87	1,00
UBC_HUMAN	Polyubiquitin C UBC 1 3	285,48	-	1,26	0,23	0,76	2,75	1,01	1,00	2,18	0,78	1,00
POTEE_HUMAN	POTE ankyrin domain family member E POTEE 1 3	19659,09	-	0,85	-0,16	0,00	1,03	0,03	0,75	1,21	0,19	1,00
POTEF_HUMAN	POTE ankyrin domain family member F POTEF 1 2	17251,10	-	0,85	-0,16	0,00	1,03	0,03	0,78	1,21	0,19	1,00
POTEI_HUMAN	POTE ankyrin domain family member I POTEI 3 1	5723,06	-	0,85	-0,16	0,00	1,03	0,03	0,69	1,21	0,19	1,00
POTEJ_HUMAN	POTE ankyrin domain family member J POTEJ 3 1	4741,38	-	0,72	-0,33	0,00	1,07	0,07	0,93	1,49	0,40	1,00
SAP_HUMAN	Proactivator polypeptide PSAP 1 2	307,40	AC	AC	AC	AC	AC	AC	AC	-	-	-
PGAM4_HUMAN	Probable phosphoglycerate mutase 4 PGAM4 2 1	3192,63	-	1,09	0,09	0,77	0,94	-0,06	0,31	0,86	-0,15	0,19
PROF1_HUMAN	Profilin 1 PFN1 1 2	40424,47	-	1,08	0,08	0,96	1,09	0,09	0,97	1,01	0,01	0,58
ANKH_HUMAN	Progressive ankylosis protein homolog ANKH 1 2	234,47	CTR	CTR	CTR	CTR	-	-	-	CTR	CTR	CTR
PSA4_HUMAN	Proteasome subunit alpha type 4 PSMA4 1 1	664,76	-	1,16	0,15	0,59	AC	AC	AC	CTR	CTR	CTR
PSB10_HUMAN	Proteasome subunit beta type 10 PSMB10 1 1	289,84	-	AC	AC	AC	0,39	-0,95	0,07	-	-	-
PDIA3_HUMAN	Protein disulfide isomerase A3 PDIA3 1 4	2031,94	-	0,76	-0,27	0,02	1,40	0,34	0,99	1,84	0,61	1,00
PDIA4_HUMAN	Protein disulfide isomerase A4 PDIA4 1 2	323,51	AC	AC	AC	AC	AC	AC	AC	-	-	-
PDIA6_HUMAN	Protein disulfide isomerase A6 PDIA6 1 1	2627,90	-	AC	AC	AC	0,90	-0,10	0,35	HAM/TSP	HAM/TSP	HAM/TSP
PDIA1_HUMAN	Protein disulfide isomerase P4HB 1 3	876,07	-	0,91	-0,09	0,35	1,08	0,08	0,67	1,19	0,17	0,74
PARK7_HUMAN	Protein DJ 1 PARK7 1 2	2045,44	-	AC	AC	AC	0,76	-0,28	0,06	HAM/TSP	HAM/TSP	HAM/TSP

S10AB_HUMAN	Protein S100 A11 S100A11 1 2	2409,08	-	AC	AC	AC	0,96	-0,04	0,42	HAM/TSP	HAM/TSP	HAM/TSP
S10A4_HUMAN	Protein S100 A4 S100A4 1 1	2332,85	-	0,45	-0,80	0,06	0,30	-1,20	0,01	0,67	-0,40	0,28
S10A6_HUMAN	Protein S100 A6 S100A6 1 1	834,08	-	AC	AC	AC	2,27	0,82	1,00	HAM/TSP	HAM/TSP	HAM/TSP
S10A8_HUMAN	Protein S100 A8 S100A8 1 1	26750,96	-	0,99	-0,01	0,42	0,25	-1,39	0,00	0,25	-1,38	0,00
S10A9_HUMAN	Protein S100 A9 S100A9 1 1	55758,54	-	1,01	0,01	0,56	1,34	0,29	1,00	1,32	0,28	1,00
PNPH_HUMAN	Purine nucleoside phosphorylase PNP 1 2	364,51	-	AC	AC	AC	0,82	-0,20	0,38	HAM/TSP	HAM/TSP	HAM/TSP
AXA2L_HUMAN	Putative annexin A2 like protein ANXA2P2 5 2	2336,99	-	1,14	0,13	0,85	1,39	0,33	0,95	1,23	0,21	0,89
ACTBM_HUMAN	Putative beta actin like protein 3 POTEKP 5 1	14461,31	-	0,76	-0,28	0,00	0,12	-2,12	0,00	0,16	-1,85	0,00
EF1A3_HUMAN	Putative elongation factor 1 alpha like 3 EEF1A1P5 5 1	817,18	-	1,77	0,57	1,00	1,52	0,42	0,95	0,86	-0,15	0,28
HSP77_HUMAN	Putative heat shock 70 kDa protein 7 HSPA7 5 2	1656,09	-	0,79	-0,23	0,15	0,35	-1,06	0,00	0,43	-0,84	0,00
HS905_HUMAN	Putative heat shock protein HSP 90 alpha A5 HSP90AA5P 1 1	235,07	HAM/TSP	-	-	-	HAM/TSP	HAM/TSP	HAM/TSP	HAM/TSP	HAM/TSP	HAM/TSP
H90B3_HUMAN	Putative heat shock protein HSP 90 beta 3 HSP90AB3P 5 1	181,82	-	-	-	-	HAM/TSP	HAM/TSP	HAM/TSP	0,83	-0,19	0,33
HGB1A_HUMAN	Putative high mobility group protein B1 like 1 HMGB1P1 5 1	849,76	HAM/TSP	-	-	-	HAM/TSP	HAM/TSP	HAM/TSP	HAM/TSP	HAM/TSP	HAM/TSP
NDK8_HUMAN	Putative nucleoside diphosphate kinase NME2P1 5 1	1351,44	-	AC	AC	AC	1,40	0,34	0,90	HAM/TSP	HAM/TSP	HAM/TSP
MSS51_HUMAN	Putative protein MSS51 homolog mitochondrial MSS51 2 2	325,94	AC	AC	AC	AC	AC	AC	AC	-	-	-
ZN321_HUMAN	Putative protein ZNF321 ZNF321P 5 3	472,07	HAM/TSP	-	-	-	HAM/TSP	HAM/TSP	HAM/TSP	HAM/TSP	HAM/TSP	HAM/TSP
RB43L_HUMAN	Putative Rab 43 like protein ENSP00000330714 5 3	364,58	AC	AC	AC	AC	AC	AC	AC	-	-	-
RAB1C_HUMAN	Putative Ras related protein Rab 1C RAB1C 5 2	1762,03	-	0,89	-0,12	0,28	0,52	-0,65	0,04	0,59	-0,53	0,11

TCP2L_HUMAN	Putative t complex protein 10A homolog 2 TCP10L2 5 2	240,31	AC	AC	AC	AC	AC	AC	AC	-	-	-
CA211_HUMAN	Putative uncharacterized protein encoded by LINC00337 LINC00337 2 1	323,79	HAM/TSP	-	-	-	HAM/TSP	HAM/TSP	HAM/TSP	HAM/TSP	HAM/TSP	HAM/TSP
KPYM_HUMAN	Pyruvate kinase isozymes M1 M2 PKM 1 4	6790,59	-	1,09	0,09	0,95	0,34	-1,07	0,00	0,31	-1,16	0,00
KPYR_HUMAN	Pyruvate kinase isozymes R L PKLR 1 2	629,40	-	1,03	0,03	0,58	0,32	-1,15	0,07	0,31	-1,18	0,07
GDIA_HUMAN	Rab GDP dissociation inhibitor alpha GDI1 1 2	1564,81	-	0,95	-0,05	0,37	0,78	-0,25	0,12	0,82	-0,20	0,25
GDIB_HUMAN	Rab GDP dissociation inhibitor beta GDI2 1 2	1963,55	-	1,08	0,08	0,71	0,94	-0,06	0,37	0,87	-0,14	0,15
RADI_HUMAN	Radixin RDX 1 1	572,32	-	1,17	0,16	0,92	0,96	-0,04	0,34	0,81	-0,21	0,03
RAC1_HUMAN	Ras related C3 botulinum toxin substrate 1 RAC1 1 1	984,60	-	2,23	0,80	0,99	3,25	1,18	0,99	1,48	0,39	0,84
RAC2_HUMAN	Ras related C3 botulinum toxin substrate 2 RAC2 1 1	1524,75	-	2,32	0,84	1,00	1,68	0,52	0,88	0,72	-0,33	0,19
RAC3_HUMAN	Ras related C3 botulinum toxin substrate 3 RAC3 1 1	804,76	-	1,48	0,39	0,87	1,63	0,49	0,78	1,11	0,10	0,60
RAB10_HUMAN	Ras related protein Rab 10 RAB10 1 1	364,58	-	0,50	-0,69	0,20	-	-	-	CTR	CTR	CTR
RAB15_HUMAN	Ras related protein Rab 15 RAB15 1 1	803,40	-	AC	AC	AC	0,19	-1,67	0,01	HAM/TSP	HAM/TSP	HAM/TSP
RAB1A_HUMAN	Ras related protein Rab 1A RAB1A 1 3	1224,79	-	0,90	-0,10	0,37	0,49	-0,72	0,01	0,53	-0,63	0,05
RAB1B_HUMAN	Ras related protein Rab 1B RAB1B 1 1	947,85	-	0,90	-0,10	0,36	0,53	-0,63	0,03	0,59	-0,53	0,08
RAB35_HUMAN	Ras related protein Rab 35 RAB35 1 1	2276,71	-	0,76	-0,27	0,32	AC	AC	AC	CTR	CTR	CTR
RAB37_HUMAN	Ras related protein Rab 37 RAB37 1 3	364,58	AC	AC	AC	AC	AC	AC	AC	-	-	-
RAB43_HUMAN	Ras related protein Rab 43 RAB43 1 1	364,58	AC	AC	AC	AC	AC	AC	AC	-	-	-
RAB7A_HUMAN	Ras related protein Rab 7a RAB7A 1 1	774,54	-	0,57	-0,57	0,24	AC	AC	AC	CTR	CTR	CTR
RAB8A_HUMAN	Ras related protein Rab 8A RAB8A 1 1	364,58	-	0,50	-0,69	0,22	AC	AC	AC	CTR	CTR	CTR

RAB8B_HUMAN	Ras related protein Rab 8B RAB8B 1 2	364,58	AC	AC	AC	AC	AC	AC	AC	-	-	-
RAP1A_HUMAN	Ras related protein Rap 1A RAP1A 1 1	612,97	-	AC	AC	AC	0,73	-0,31	0,27	HAM/TSP	HAM/TSP	HAM/TSP
RAP1B_HUMAN	Ras related protein Rap 1b RAP1B 1 1	903,61	-	AC	AC	AC	0,88	-0,13	0,38	HAM/TSP	HAM/TSP	HAM/TSP
RP1BL_HUMAN	Ras related protein Rap 1b like protein 2 1	497,24	HAM/TSP	-	-	-	HAM/TSP	HAM/TSP	HAM/TSP	HAM/TSP	HAM/TSP	HAM/TSP
RSU1_HUMAN	Ras suppressor protein 1 RSU1 1 3	2856,20	-	0,88	-0,13	0,12	0,87	-0,14	0,15	0,99	-0,01	0,45
RTN1_HUMAN	Reticulon 1 RTN1 1 1	347,72	HAM/TSP	-	-	-	HAM/TSP	HAM/TSP	HAM/TSP	HAM/TSP	HAM/TSP	HAM/TSP
RTN4_HUMAN	Reticulon 4 RTN4 1 2	217,40	HAM/TSP	-	-	-	HAM/TSP	HAM/TSP	HAM/TSP	HAM/TSP	HAM/TSP	HAM/TSP
GDIR1_HUMAN	Rho GDP dissociation inhibitor 1 ARHGDIA 1 3	3583,26	HAM/TSP	-	-	-	HAM/TSP	HAM/TSP	HAM/TSP	HAM/TSP	HAM/TSP	HAM/TSP
GDIR2_HUMAN	Rho GDP dissociation inhibitor 2 ARHGDIB 1 3	4059,44	-	1,63	0,49	1,00	1,34	0,29	0,98	0,82	-0,20	0,08
RHOC_HUMAN	Rho related GTP binding protein RhoC RHOC 1 1	549,24	-	0,99	-0,01	0,47	1,57	0,45	0,84	1,57	0,45	0,95
RHOG_HUMAN	Rho related GTP binding protein RhoG RHOG 1 1	681,36	HAM/TSP	-	-	-	HAM/TSP	HAM/TSP	HAM/TSP	HAM/TSP	HAM/TSP	HAM/TSP
RHOJ_HUMAN	Rho related GTP binding protein RhoJ RHOJ 2 1	638,38	HAM/TSP	-	-	-	HAM/TSP	HAM/TSP	HAM/TSP	HAM/TSP	HAM/TSP	HAM/TSP
RHOQ_HUMAN	Rho related GTP binding protein RhoQ RHOQ 1 2	655,29	HAM/TSP	-	-	-	HAM/TSP	HAM/TSP	HAM/TSP	HAM/TSP	HAM/TSP	HAM/TSP
RINI_HUMAN	Ribonuclease inhibitor RNH1 1 2	1240,70	-	CTR	CTR	CTR	HAM/TSP	HAM/TSP	HAM/TSP	1,25	0,22	0,84
ESTD_HUMAN	S formylglutathione hydrolase ESD 1 2	1253,40	-	1,23	0,21	0,73	0,64	-0,45	0,12	0,52	-0,66	0,01
SEPT2_HUMAN	Septin 2 SEPT2 1 1	548,06	HAM/TSP	-	-	-	HAM/TSP	HAM/TSP	HAM/TSP	HAM/TSP	HAM/TSP	HAM/TSP
SEPT6_HUMAN	Septin 6 SEPT6 1 4	338,96	HAM/TSP	-	-	-	HAM/TSP	HAM/TSP	HAM/TSP	HAM/TSP	HAM/TSP	HAM/TSP
PP1B_HUMAN	Serine threonine protein phosphatase PP1 beta catalytic subunit PPP1CB 1 3	399,51	HAM/TSP	-	-	-	HAM/TSP	HAM/TSP	HAM/TSP	HAM/TSP	HAM/TSP	HAM/TSP

ALBU_BOVIN	Serum albumin Bos taurus ALB 1 4	8114,12	-	0,47	-0,75	0,00	2,12	0,75	1,00	4,48	1,50	1,00
ALBU_HUMAN	Serum albumin ALB 1 2	1732,98	-	1,04	0,04	0,70	1,07	0,07	0,79	1,03	0,03	0,61
SDPR_HUMAN	Serum deprivation response protein SDPR 1 3	710,10	HAM/TSP	-	-	-	HAM/TSP	HAM/TSP	HAM/TSP	HAM/TSP	HAM/TSP	HAM/TSP
SH3L2_HUMAN	SH3 domain binding glutamic acid rich like protein 2 SH3BGRL2 1 2	2511,77	HAM/TSP	-	-	-	HAM/TSP	HAM/TSP	HAM/TSP	HAM/TSP	HAM/TSP	HAM/TSP
SH3L3_HUMAN	SH3 domain binding glutamic acid rich like protein 3 SH3BGRL3 1 1	3530,08	-	AC	AC	AC	2,25	0,81	1,00	HAM/TSP	HAM/TSP	HAM/TSP
SH3L1_HUMAN	SH3 domain binding glutamic acid rich like protein SH3BGRL 1 1	485,98	-	CTR	CTR	CTR	HAM/TSP	HAM/TSP	HAM/TSP	0,89	-0,12	0,33
GTR12_HUMAN	Solute carrier family 2 facilitated glucose transporter member 12 SLC2A12 2 1	236,93	HAM/TSP	-	-	-	HAM/TSP	HAM/TSP	HAM/TSP	HAM/TSP	HAM/TSP	HAM/TSP
SNX4_HUMAN	Sorting nexin 4 SNX4 1 1	313,47	HAM/TSP	-	-	-	HAM/TSP	HAM/TSP	HAM/TSP	HAM/TSP	HAM/TSP	HAM/TSP
SPRC_HUMAN	SPARC SPARC 1 1	992,13	HAM/TSP	-	-	-	HAM/TSP	HAM/TSP	HAM/TSP	HAM/TSP	HAM/TSP	HAM/TSP
SPAT1_HUMAN	Spermatogenesis associated protein 1 SPATA1 2 3	316,46	HAM/TSP	-	-	-	HAM/TSP	HAM/TSP	HAM/TSP	HAM/TSP	HAM/TSP	HAM/TSP
SRC8_HUMAN	Src substrate cortactin CTTN 1 2	352,84	HAM/TSP	-	-	-	HAM/TSP	HAM/TSP	HAM/TSP	HAM/TSP	HAM/TSP	HAM/TSP
STMN1_HUMAN	Stathmin STMN1 1 3	1184,97	HAM/TSP	-	-	-	HAM/TSP	HAM/TSP	HAM/TSP	HAM/TSP	HAM/TSP	HAM/TSP
GRP75_HUMAN	Stress 70 protein mitochondrial HSPA9 1 2	231,36	HAM/TSP	-	-	-	HAM/TSP	HAM/TSP	HAM/TSP	HAM/TSP	HAM/TSP	HAM/TSP
SODC_HUMAN	Superoxide dismutase Cu Zn SOD1 1 2	2309,96	HAM/TSP	-	-	-	HAM/TSP	HAM/TSP	HAM/TSP	HAM/TSP	HAM/TSP	HAM/TSP
TLN1_HUMAN	Talin 1 TLN1 1 3	8703,68	-	0,74	-0,30	0,00	1,36	0,31	1,00	1,84	0,61	1,00
TLN2_HUMAN	Talin 2 TLN2 1 4	219,33	-	-	-	-	1,08	0,08	0,66	HAM/TSP	HAM/TSP	HAM/TSP
TSYL4_HUMAN	Testis specific Y encoded like protein 4 TSPYL4 2 2	292,98	CTR	CTR	CTR	CTR	-	-	-	CTR	CTR	CTR
THIO_HUMAN	Thioredoxin TXN 1 3	1902,87	HAM/TSP	-	-	-	HAM/TSP	HAM/TSP	HAM/TSP	HAM/TSP	HAM/TSP	HAM/TSP

TSP1_HUMAN	Thrombospondin 1 THBS1 1 2	4132,85	-	0,94	-0,06	0,20	1,14	0,13	0,95	1,21	0,19	0,99
TSP2_HUMAN	Thrombospondin 2 THBS2 1 2	223,66	-	CTR	CTR	CTR	HAM/TSP	HAM/TSP	HAM/TSP	3,49	1,25	1,00
TYPH_HUMAN	Thymidine phosphorylase TYMP 1 2	1753,43	-	0,81	-0,21	0,30	AC	AC	AC	CTR	CTR	CTR
TYB4_HUMAN	Thymosin beta 4 TMSB4X 1 2	13165,78	-	0,15	-1,87	0,00	1,43	0,36	1,00	9,30	2,23	1,00
TALDO_HUMAN	Transaldolase TALDO1 1 2	1371,56	-	0,92	-0,08	0,28	0,95	-0,05	0,36	1,03	0,03	0,55
RHOA_HUMAN	Transforming protein RhoA RHOA 1 1	593,30	-	0,96	-0,04	0,43	1,62	0,48	0,91	1,68	0,52	0,98
TAGL2_HUMAN	Transgelin 2 TAGLN2 1 3	5598,13	-	0,90	-0,11	0,13	1,32	0,28	1,00	1,46	0,38	1,00
TRPV6_HUMAN	Transient receptor potential cation channel subfamily V member 6 TRPV6 1 2	249,22	HAM/TSP	-	-	-	HAM/TSP	HAM/TSP	HAM/TSP	HAM/TSP	HAM/TSP	HAM/TSP
TERA_HUMAN	Transitional endoplasmic reticulum ATPase VCP 1 4	2724,94	-	0,96	-0,04	0,40	0,68	-0,38	0,01	0,72	-0,33	0,05
TKT_HUMAN	Transketolase TKT 1 3	1747,40	-	0,72	-0,33	0,00	0,79	-0,23	0,01	1,09	0,09	0,79
TPIS_HUMAN	Triosephosphate isomerase TPI1 1 3	7742,80	-	0,96	-0,04	0,35	1,31	0,27	1,00	1,36	0,31	0,97
TMOD3_HUMAN	Tropomodulin 3 TMOD3 1 1	469,26	AC	AC	AC	AC	AC	AC	AC	-	-	-
TPM1_HUMAN	Tropomyosin alpha 1 chain TPM1 1 2	1164,26	-	0,40	-0,91	0,00	0,97	-0,03	0,42	2,41	0,88	1,00
TPM3_HUMAN	Tropomyosin alpha 3 chain TPM3 1 2	3437,67	-	0,36	-1,01	0,00	0,94	-0,06	0,34	2,59	0,95	1,00
TPM4_HUMAN	Tropomyosin alpha 4 chain TPM4 1 3	2615,34	-	0,68	-0,38	0,02	1,54	0,43	1,00	2,27	0,82	1,00
TPM2_HUMAN	Tropomyosin beta chain TPM2 1 1	1706,43	-	0,39	-0,94	0,00	0,18	-1,74	0,00	0,45	-0,80	0,00
SYWC_HUMAN	Tryptophan tRNA ligase cytoplasmic WARS 1 2	573,06	HAM/TSP	-	-	-	HAM/TSP	HAM/TSP	HAM/TSP	HAM/TSP	HAM/TSP	HAM/TSP
TBG2_HUMAN	Tubulin gamma 2 chain TUBG2 1 1	277,36	CTR	CTR	CTR	CTR	-	-	-	CTR	CTR	CTR
TWF2_HUMAN	Twinfilin 2 TWF2 1 2	2780,19	-	0,76	-0,28	0,23	0,95	-0,05	0,46	1,26	0,23	0,69
PTN6_HUMAN	Tyrosine protein phosphatase non receptor type 6 PTPN6 1 1	232,48	-	CTR	CTR	CTR	HAM/TSP	HAM/TSP	HAM/TSP	1,04	0,04	0,49

RS27A_HUMAN	Ubiquitin 40S ribosomal protein S27a RPS27A 1 2	1098,63	-	1,04	0,04	0,51	2,41	0,88	0,99	2,32	0,84	0,99
RL40_HUMAN	Ubiquitin 60S ribosomal protein L40 UBA52 1 2	1098,63	-	1,52	0,42	0,81	3,60	1,28	1,00	2,36	0,86	1,00
UB2L3_HUMAN	Ubiquitin conjugating enzyme E2 L3 UBE2L3 1 1	1247,25	AC	AC	AC	AC	AC	AC	AC	-	-	-
UBA1_HUMAN	Ubiquitin like modifier activating enzyme 1 UBA1 1 3	305,30	-	1,13	0,12	0,73	1,09	0,09	0,72	0,98	-0,02	0,43
CG026_HUMAN	Uncharacterized protein C7orf26 C7orf26 2 1	258,79	HAM/TSP	-	-	-	HAM/TSP	HAM/TSP	HAM/TSP	HAM/TSP	HAM/TSP	HAM/TSP
VASP_HUMAN	Vasodilator stimulated phosphoprotein VASP 1 3	653,43	-	AC	AC	AC	2,10	0,74	1,00	HAM/TSP	HAM/TSP	HAM/TSP
VIME_HUMAN	Vimentin VIM 1 4	332,75	-	AC	AC	AC	0,45	-0,79	0,01	-	-	-
VINC_HUMAN	Vinculin VCL 1 4	3800,98	-	0,79	-0,24	0,00	0,93	-0,07	0,26	1,19	0,17	0,94
WDR1_HUMAN	WD repeat containing protein 1 WDR1 1 4	1063,59	-	AC	AC	AC	0,64	-0,45	0,04	HAM/TSP	HAM/TSP	HAM/TSP
XPP1_HUMAN	Xaa Pro aminopeptidase 1 XPNPEP1 1 3	425,25	HAM/TSP	-	-	-	HAM/TSP	HAM/TSP	HAM/TSP	HAM/TSP	HAM/TSP	HAM/TSP
XYLB_HUMAN	Xylulose kinase XYLB 1 3	218,58	CTR	CTR	CTR	CTR	-	-	-	CTR	CTR	CTR
Z512B_HUMAN	Zinc finger protein 512B ZNF512B 1 1	217,01	HAM/TSP	-	-	-	HAM/TSP	HAM/TSP	HAM/TSP	HAM/TSP	HAM/TSP	HAM/TSP
ZYX_HUMAN	Zyxin ZYX 1 1	1687,84	-	0,64	-0,45	0,01	1,54	0,43	1,00	2,41	0,88	1,00

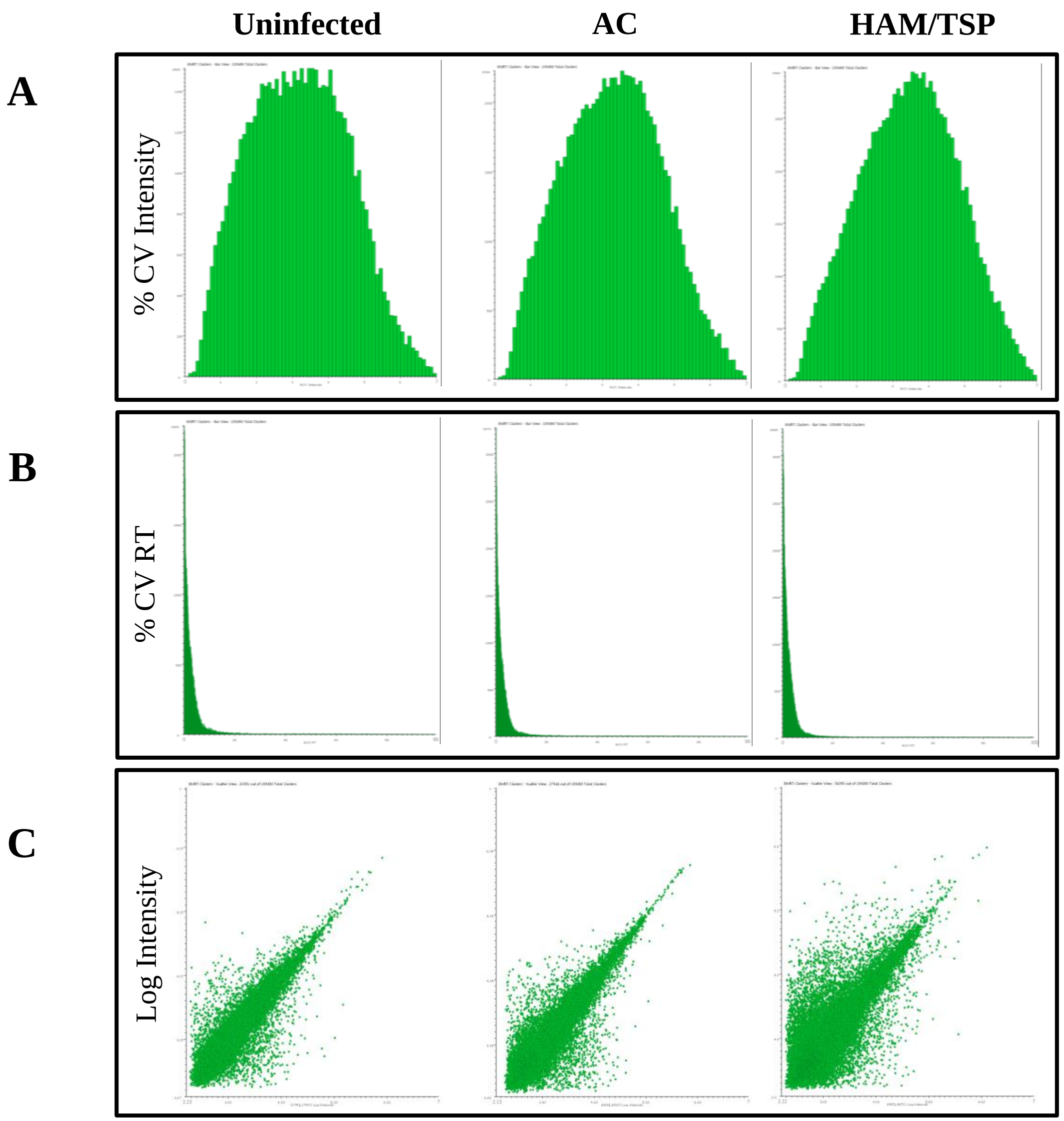

Figure S1. Statistical analysis of the LCMS^E replicate experiments. (A) Coefficients of variation for the identified clusters. (B) Coefficients of variation for the retention times in all analyses were centered at 4% (C) Representative binary comparison of the log intensity obtained from two replicates of the three injections.

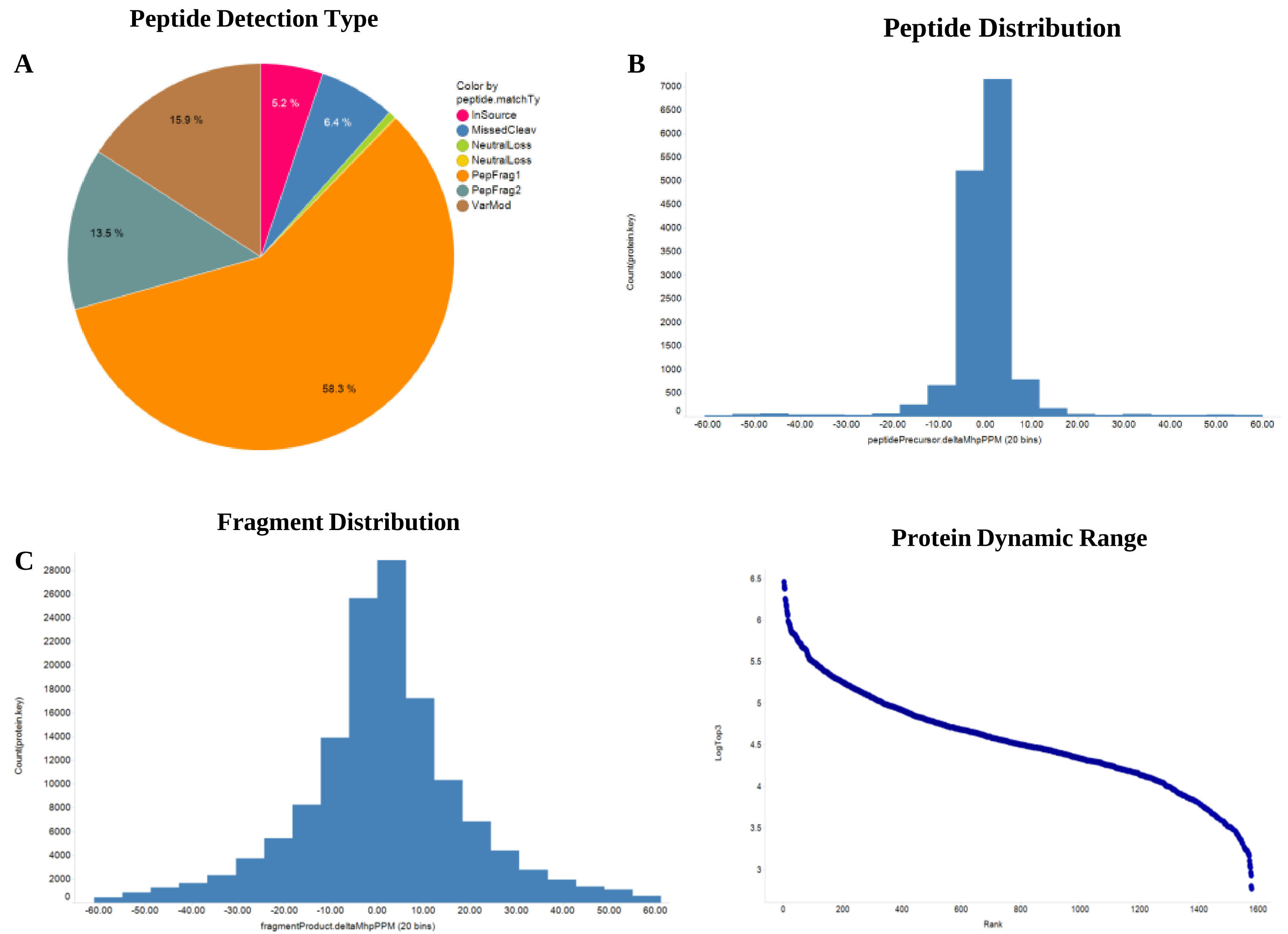

Figure S2. Data control quality of all replicates. (A) Peptide detection type was verified by peptide match analysis to confirm the quality of fragmentation and the trypsin digestion (B) Distribution of fragment products for approximately 75% of the fragment ions in a 15 ppm error range. (C) Experiment ppm error for precursor ion within 10 ppm mass range was estimated in 88%, whereas fragment ions were estimated in 77% within 20 ppm mass range. (D) Protein dynamic range of all replicate samples. The graph represents identified protein rank by log10Top3. Top3 means the summed intensity of the three most intense precursor ions from the corresponding protein. Nearly four orders of magnitude are observed. To normalize the samples, we used fibrinogen γ-chain (P02679-FIBG_Human).

A

B

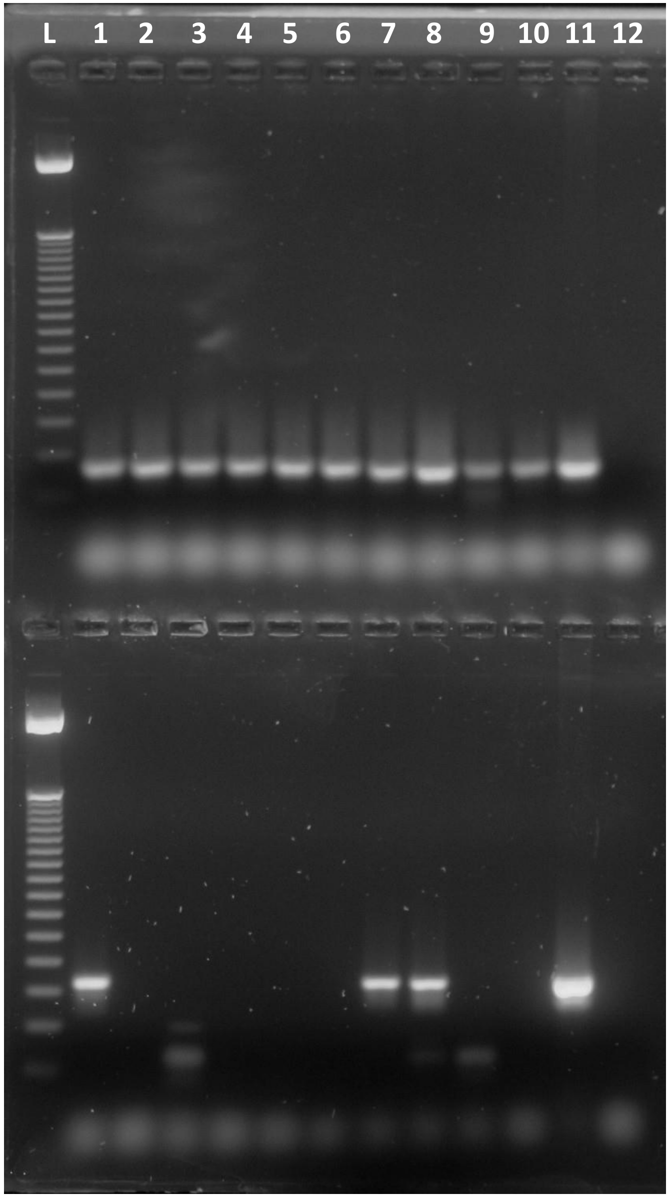

Figure S3. Full-length gels. $CD14^+$ cells were purified from PBMC derived from HTLV-1-infected patients, and DNA was extracted (Samples 1−10). HTLV-1 provirus was detect by PCR for (**B**) HTLV-1 tax gene (159bp), and amplification of (**A**) human β-globin gene (79bp) was used as internal control. DNA from the HTLV-1-infected MT2 cell line (Sample 11) and water (Sample 12) were respectively used as positive and negative controls. L= ladder.
